# Phenotellurazine redox catalysts: elements of design for radical cross-dehydrogenative coupling reactions

**DOI:** 10.3762/bjoc.20.112

**Published:** 2024-06-04

**Authors:** Alina Paffen, Christopher Cremer, Frederic W Patureau

**Affiliations:** 1 Institute of Organic Chemistry, RWTH Aachen University, Landoltweg 1, 52074 Aachen, Germany

**Keywords:** cross-dehydrogenative coupling, O_2_ activation, phenotellurazine, redox catalysis, Te catalysis

## Abstract

Redox active phenotellurazine catalysts have been recently utilized in two different cross-dehydrogenative coupling reactions. In this study, we revisit the design of the phenotellurazine redox catalysts. In particular, we investigate the level of cooperativity between the Te- and N-centers, the effect of secondary versus tertiary N-centers, the effect of heterocyclic versus non-heterocyclic structures, and the effect of substitution patterns on the redox catalytic activity.

## Introduction

**Scheme 1:**
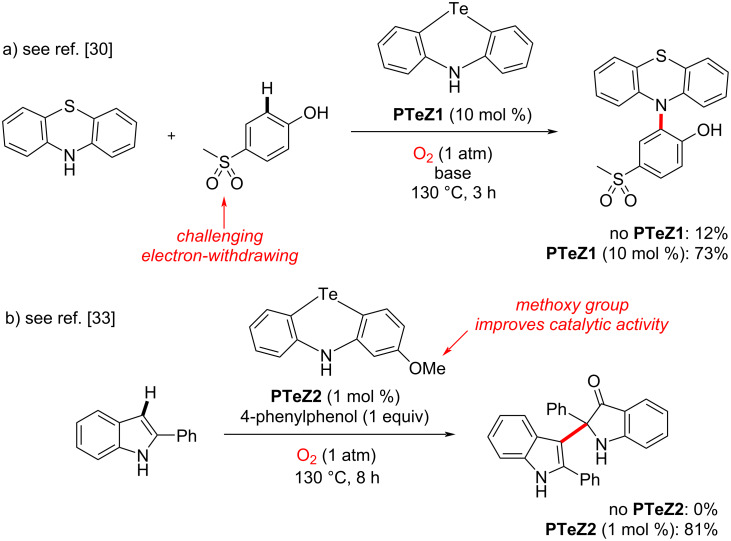
Phenotellurazine-catalyzed cross-dehydrogenative couplings.

Tellurium catalysis has become increasingly important in recent years. This is due to its unique chalcogen bonding ability, thus enabling the activation of small yet highly relevant organic substrates. For example, Huber and co-authors recently designed a Te-based catalyst in an indole Michael addition reaction [1–5]. Pale and Mamane utilized another Te-based catalyst in an electrophilic bromine-mediated cyclization reaction [6–7], and Gabbaï yet another in a different cyclization reaction [8–9], among other catalytic chalcogen bonding activation examples [10–29]. In contrast, we have reported recently some redox-active Te-based catalysts, which exploit the redox flexibility of tellurium, especially in the context of phenotellurazine scaffolds. Notably, we showed that phenotellurazine **PTeZ1** could significantly catalyze the cross-dehydrogenative phenothiazination of phenols bearing challenging electron-withdrawing substituents under a simple oxygen atmosphere (Scheme 1a) [30–32]. Most recently, we also showed that phenotellurazines could catalyze the oxidative dimerization of indoles, likewise under a simple oxygen atmosphere. 2-Methoxyphenotellurazine **PTeZ2** proved to be the optimal catalyst in the latter case (Scheme 1b) [33].

In the present study, we decided to revisit the design of the phenotellurazine redox catalyst, in the hope of improving it as well as enabling new catalytic reactivity. In particular, we wished to investigate and optimize the level of electronic cooperativity between the Te- and N-centers, the effect of secondary versus tertiary N-centers, the effect of heterocyclic versus non-heterocyclic structures, and the effect of various substitution patterns.

## Results and Discussion

With this aim in mind, we thus started investigating Te(II) redox catalyst candidates that do not necessarily carry a N-atom in the structure, or else at different positions, in order to establish how their redox catalytic reactivity might be affected. Indeed, we learned recently that amino-arenes possess some level of redox catalytic activity by themselves, in the absence of a Te-center [34], and therefore wished to investigate and optimize their redox contribution within the Te(II) catalysts.

**Scheme 2:**
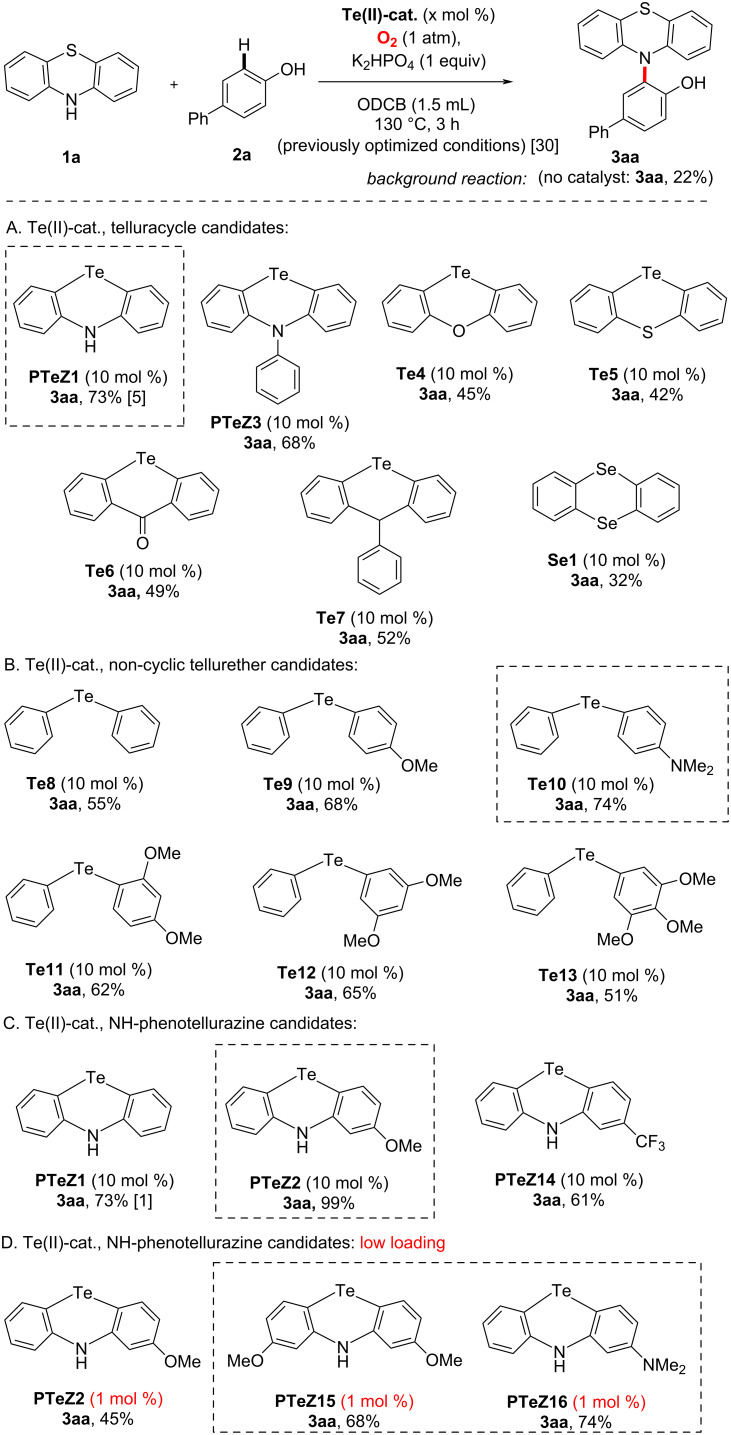
Screening of new Te(II)-catalyst candidates. ODCB: *ortho*-dichlorobenzene.

Multiple synthetic efforts were therefore deployed in order to access several key Te(II) targets, both with and without N-functional groups, in heterocyclic as well as in non-heterocyclic fashion. These were then investigated in the Te(II)-catalyzed benchmark dehydrogenative phenothiazination of phenols, a reaction that we discovered in 2015 [35–37], under analogous conditions as previously described [30–32]. The results are summarized in Scheme 2. The multistep synthesis and characterization of all Te-based catalyst candidates can be found in Supporting Information File 1. In the main article we will focus on catalysis results.

We therefore first tested various telluracycle candidates, in particular without an NH bridge (catalyst candidates **PTeZ3**, **Te4**–**Te7**, Scheme 2A). These telluracycles, bridged by very diverse electron-donating or -withdrawing functional groups all featured significantly inferior catalytic activity in the selected benchmark reaction (O_2_-mediated dehydrogenative phenothiazination of 4-phenylphenol). It can therefore be deduced that the N-bridging atom is important for catalytic activity. In order to further investigate the matter, we next turned our attention to non-heterocyclic tellurethers **Te8**–**Te13**, bearing various substituents (Scheme 2B). Expectedly, simple diphenyl tellurether **Te8** featured degraded catalytic activity (**3aa**, 55% after 3 h), although this still represents a significantly superior result compared to catalyst-free conditions (**3aa**, 22% after 3 h). Electron π-donating substituents considerably improved catalytic activity, with an optimum among tested structures for **Te10** (**3aa**, 74% after 3 h), which is intriguingly similar to the previously reported best catalyst for this reaction: **PTeZ1** (**3aa**, 73% after 3 h). In other words, the cyclic phenotellurazine character of the catalyst does not seem to be essential in the context of this particular reaction, as opposed to the *ortho/para* substitution pattern of Te- and N-atoms. Nevertheless, all results considered, we elected at this point to keep the cyclic phenotellurazine structure of the catalyst in the hope of increased catalytic robustness, especially in view of further increasing substitution. Next, we therefore tested methoxy-substituted **PTeZ2**, a successful catalyst structure which we recently developed for the cross-dehydrogenative coupling of indoles [33], in the same benchmark reaction. To our satisfaction, **PTeZ2** proved to be the most active catalyst so far in this study (**3aa**, 99% after 3 h). In order to further optimize the catalyst structure, we then reduced the catalytic loading by one order of magnitude, from the standard 10 mol % to only 1 mol %, all other reaction parameters remaining identical. In these considerably more demanding conditions, **PTeZ2** still performed admirably well (**3aa**, 45% at 3 h), significantly better than the non-catalyzed reaction (**3aa**, 22% after 3 h). Going from 2-methoxy towards 2,8-dimethoxy substitution (**PTeZ15**) allowed to significantly increase catalytic activity (**3aa**, 68% after 3 h), still at 1 mol % loading. Encouraged by this trend, which seemed to indicate that the more π-electron-donating substituents increase catalytic activity, we continued structural optimization. Thus, 2-dimethylamino-substituted **PTeZ16** performed even better (**3aa**, 74% after 3 h and 1 mol % loading).

**Scheme 3:**
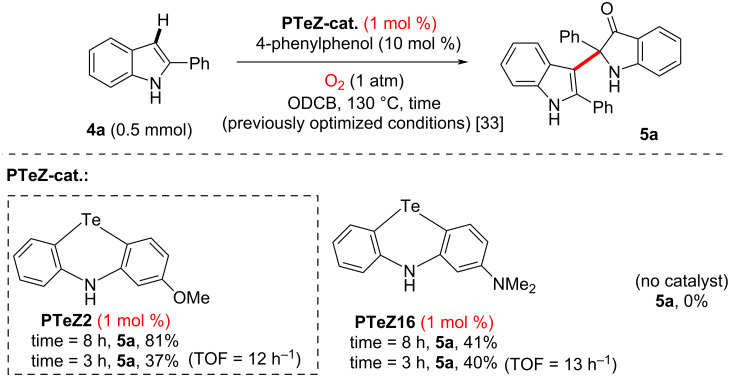
Phenotellurazine-catalyzed cross-dehydrogenative indole dimerization.

In order to evaluate these new Te-catalysts further, we then turned our attention to the second, arguably more challenging test reaction (Scheme 3). In particular, we hoped that the more π-electron-rich optimized **PTeZ16** (2-dimethylamino substituent) would outperform previously published **PTeZ2** (2-methoxy) in that particular reaction. Unfortunately, this was not the case. At 1 mol % catalytic loading and 8 h reaction time, **PTeZ2** considerably outperforms **PTeZ16** (indole **4a** towards product **5a**, 81 versus 41%, respectively). Intrigued by these results, we also checked yields of product **5a** at only 3 h reaction time for both catalysts. Thus, at shorter reaction time, it transpires that **PTeZ2** and **PTeZ16** perform similarly overall (**5a**, 37 versus 40%, respectively). It can therefore be concluded that while **PTeZ16** (2-dimethylamino) shows promising initial catalytic activity, it is not robust enough to survive the oxidative high temperature conditions for a prolonged period of time. In contrast, as was previously demonstrated in the literature, **PTeZ2** (2-methoxy substituent) features a far greater chemical stability, to such an extent that it could be in large part recovered at the end of the reaction (see previous study) [33]. In other words, **PTeZ2** (2-methoxy substituent) features the best compromise in terms of electronic effects, which affect both the stability and reactivity of the key catalytically active intermediate(s), possibly including chalcogen bonding activation ability of the substrates [38].

## Conclusion

In conclusion, we demonstrated the importance of the phenotellurazine scaffold, bearing both a Te(II) center as well as a N-bridge, for redox catalytic activity. However, although the idea of increasing the π-electron-rich character of the phenotellurazine catalyst had seemed very promising at first, our results show that this strategy leads to overall less robust Te(II) catalysts under oxidative reaction conditions. This in turn generally leads to inferior catalytic performance. Our future research efforts in the area of Te(II) catalysis will likely focus on milder coupling reactions on the one hand, and/or on novel more robust and more active ligand designs on the other. In particular, more investigations will likely be needed regarding the optimization of the possible Te-substrate interaction.

## Supporting Information

File 1 Experimental section and characterization of synthesized compounds.

## Data Availability

All data that supports the findings of this study is available in the published article and/or the supporting information to this article.
