# Supplementary material for: Phenotellurazine redox catalysts: elements of design for radical cross-dehydrogenative coupling reactions
Source: Beilstein J Org Chem. 2024 Jun 4;20:1292–7. doi: 10.3762/bjoc.20.112 (PMC11181166; doi:10.3762/bjoc.20.112)

BEILSTEIN JOURNAL OF ORGANIC CHEMISTRY

Supporting Information

for

Phenotellurazine redox catalysts: elements of design for radical cross-dehydrogenative coupling reactions

Alina Paffen, Christopher Cremer and Frederic W. Patureau

Beilstein J. Org. Chem. **2024,** *20,* 1292–1297. doi:10.3762/bjoc.20.112

Experimental section and characterization of synthesized compounds

License and Terms: This is a supporting information file under the terms of the Creative Commons Attribution License (https://creativecommons.org/licenses/by/4.0). Please note that the reuse, redistribution and reproduction in particular requires that the author(s) and source are credited and that individual graphics may be subject to special legal provisions.
The license is subject to the *Beilstein Journal of Organic Chemistry* terms and conditions: (https://www.beilstein-journals.org/bjoc/terms)

Table of Contents

General information (p. S2)

Synthesis of Te and Se catalyst candidates (p. S3)

Catalytic test reaction (p. S14)

References (p. S16)

NMR Spectra (p. S17)

General Information

All commercially available chemicals were purchased from Sigma Aldrich, Alfa Aesar, BLDPharm, fluorochem, TCI, Acros organic, chemPUR or abcr and used without further purification unless stated otherwise. **PTeZ1**,[S1] **PTeZ2**,[S2] **Te4**,[S3] **PTeZ14**,[S4] bis(2-bromophenyl)sulfane,[S5] 2,2'-dibromobenzophenone,[S6] 2,2'-dibromotriphenylmethane,[S7] 4-bromo-*N,N*-dimethylamino-3-nitroaniline,[S8] 4-bromo-*N*[1],*N*[1]-dimethylbenzene-1,3-diamine,[S9] 4-Iodo-*N,N*-dimethylaniline[S10] and other precursors[S11-S20] were synthesized according to literature procedures. **PTeZ3** was prepared according to a known procedure.[S21]
Analytical thin-layer chromatography (TLC) was performed with silica gel plates. The products were visualized by UV detection at a wavelength of 254 nm. UV inactive substances were visualized using iodine, potassium permanganate ($KMnO_4$) or vanillin stain. Unless stated otherwise, column chromatography was performed using silica gel (0.04–0.063 nm) as filling material. The solvent ratios are specified in volume shares. NMR analysis was performed at room temperature on a Varian V-NMRS 400, V-NMRS 600, Varian Mercury 300, Bruker Avance 400 or Bruker Avance 600. Deuterated chloroform ($CDCl_3$) or deuterated dimethyl sulfoxide (DMSO-d_6) served as solvents for NMR sample preparation. The evaluation of the obtained NMR spectra, including integration of signal areas, peak selection as well as the determination of the coupling constants, was performed using MestReNova software. Chemical shifts (δ) are specified in parts per million (ppm) relative to the residual solvent peak ($CDCl_3$: δ = 7.26 ppm, δ = 77.16 ppm; DMSO-d_6: δ = 2.50 ppm, δ = 38.52 ppm). Coupling constants (*J*) are given in Hertz (Hz). High-resolution mass spectra (HRMS) were recorded on a Thermo Scientific LTQ Orbitrap XL (ESI), Finnigan MAT95 (EI, 70 eV) or Bruker Maxis II LC-MS-System (APCI). IR spectra were measured on a PerkinElmer 100 FT-IR spectrometer with an UATR Diamond KRS-5 unit. The melting points were determined on a BüchiI Melting Point M-560 with a heating rate of 5 °C/min for Te5 & Te7 and 3 °C/min for PTeZ15 PTeZ16. The first temp. is the melting onset and the second temp. is the temperature at which the sample was fully molten or decomposed.

Safety Note: Some experiments reported here are running in an O_2 atmosphere. Standard laboratory protection should be used. We strongly recommend using a protective Plexiglas shield in front of the reactor. No explosion nor any related incident occurred while performing the experiments described below.

Synthesis of Te and Se catalyst candidates

The literature procedure for the synthesis of 10*H*-phenotellurazine (**PTeZ1**) was described by Cremer et al. in 2021 and proceeds via Buchwald–Hartwig amination,[S1] followed by an Aromatic Finkelstein reaction and the ring closing under basic conditions. We wanted to investigate if the aromatic Finkelstein reaction is necessary or if bis(2-bromophenyl)amine **SI-3** can be converted to **PTeZ1** in good yields directly. Therefore, we used the conditions described for the ring closing by Patureau and co-workers[S1] but used an elevated reaction temperature of 150 °C instead of 120 °C.

Table S1: Comparison of the yields in a two- and in a three-step synthesis of **PTeZ1**.

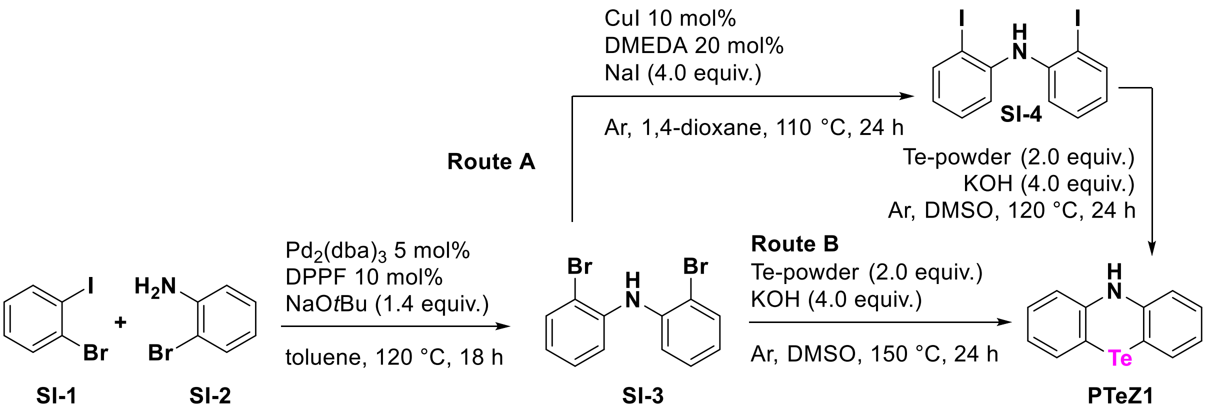

Entry	Method	Yield PTeZ1 [%]
**1**	A	64[a]
**2**	B	26

[a]: calculated *via* $Y_{\mathbf{PTeZH-1}} = Y_{\text{Aromatic Finkelstein reaction}} \cdot Y_{\text{ring closing}}$ using the yields taken from Cremer *et al.*[S1]

The results in Table S1 show that using the aromatic Finkelstein reaction is not necessary to obtain **PTeZ1** (entry 2) but the yield is more than doubled if it is applied (entry 1). Therefore, the method at an elevated temperature can be used in cases where the aromatic Finkelstein reaction did not work well and not all bromo-substituents were converted to the iodo-ones to obtain a higher yield of the resulting tellurium-based compound.

Route A is described in reference [S1]. **Route B**:

Synthesis of 10*H*-phenotellurazine PTeZ1 according to Route B

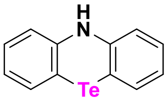

10*H*-Phenotellurazine (**PTeZ1**) was synthesized using bis(2-bromophenyl)amine as substrate and an elevated reaction temperature. Tellurium powder (1656 mg, 13.0 mmol, 2 equiv) and KOH (1471 mg, 26.0 mmol, 4.0 equiv) were weighed into a vial. The vial was sealed and then evacuated and flushed with argon for three times. Bis(2-bromophenyl)amine (2086 mg, 6.5 mmol, 1.0 equiv) and dry DMSO (5 mL) were added under argon. The reaction mixture was stirred at 150 °C for 24 h. After cooling down to room temperature, the reaction mixture was diluted with sat. NH_4Cl. The aqueous layers were extracted with DCM, the combined organic layers were dried over $MgSO_4$ and the solvent was removed in vacuo. The crude product was purified via column

chromatography using hexane/DCM in a 7:3 ratio to obtain the title compound as orange solid (491 mg, 1.7 mmol, 26%).

^{1}H-NMR (400 MHz, DMSO-*d₆*): δ = 8.52 (s, 1H), 7.32 (dd, *J* = 7.5, 1.5 Hz, 2H), 7.08 - 6.96 (m, 2H), 6.94 - 6.83 (m, 2H), 6.79 - 6.65 (m, 2H) ppm.

The NMR spectrum is in accordance with the literature values.[S1]

Synthesis of SeAn (Se1)

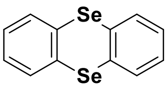

Selenium powder (392 mg, 5.0 mmol, 1.0 equiv) and KOH (1119 mg, 20.0 mmol, 4.0 equiv) were weighed into a vial. The vial was sealed and then evacuated and flushed with argon for three times. 1-Bromo-2-iodobenzene (1.28 mL, 10.0 mmol, 2.0 equiv) and dry DMSO (15 mL) were added under argon. The reaction mixture was stirred at 120 °C for 24 h. After cooling down to room temperature, the reaction mixture was diluted with sat. NH_4Cl. The aqueous layers were extracted with DCM, the combined organic layers were dried over Na_2SO_4 and the solvent was removed in vacuo. The crude product was purified via column chromatography using pure hexane to obtain the title compound as yellow solid (156 mg, 0.5 mmol, 20%).

^{1}H-NMR (400 MHz, DMSO-d_6): δ = 7.81-7.76 (second order m, 4H), 7.37-7.31 (second order m, 4H) ppm.

^{13}C-NMR (151 MHz, DMSO-d_6): δ = 133.63, 131.28, 128.45 ppm.

^{77}Se-NMR (115 MHz, DMSO-d_6): δ = 461.64 (s) ppm.

HRMS (APCI, m/z), calculated for $C_{12}H_9Se_2$ ($[M+H]^+$): 312.90292, found: 312.90296.

IR (neat, cm^{-1}): 3045, 2675, 2323, 2105, 1992, 1923, 1801, 1702, 1619, 1550, 1473, 1425, 1244, 1156, 1114, 1081, 1023, 944, 867, 745.

Synthesis of Phenoxatellurine Te4

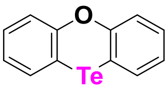

Phenoxatellurine **Te4** was synthesized according to the procedure described by Mostaghimi *et al.*[S3] Diphenyl ether (851 mg, 5.0 mmol, 1.0 equiv) and $TeCl_4$ (1.35 g, 5.0 mmol, 1.0 equiv) were weighed into a flask equipped with a reflux condenser. The mixture was heated to 150 °C for 2 h. The resulting melt was stirred at 200 °C for an additional 4 h. After cooling down to room temperature, the solid was ground, stirred in diethyl ether and filtered off. The solid was stirred in acetone and filtered off again. The solvent was removed in vacuo. The resulting brown oil was placed in a flask with $Na_2S{\cdot}xH_2O$ (3.53 g, 15 mmol, 3.0 equiv) and the mixture was stirred at 100 °C for 15 minutes. After cooling down to room temperature, the melt was washed with water and stirred in diethyl ether. The solvent was removed in vacuo and the crude product was purified via column chromatography using hexane/ethyl acetate as solvents in a 100:1 ratio. The title product was obtained as red solid (217 mg, 0.74 mmol, 15%). Note: small impurities were detected in the NMR spectra.

^{1}H-NMR (400 MHz, DMSO-d$_6$): δ = 7.67 (dd, *J* = 7.6, 1.5 Hz, 2H), 7.30 – 7.19 (m, 4H), 7.10 - 7.03 (m, 2H) ppm.

^{13}C-NMR (101 MHz, DMSO-*d$_6$*): δ = 155.49, 135.43, 129.04, 125.51, 119.16, 104.22 ppm.

^{125}Te-NMR (126 MHz, DMSO-*d$_6$*): δ = 425.49 (s) ppm.

^{1}H-NMR (300 MHz, $CDCl_3$): δ=7.55 - 7.48 (m, 2H), 7.27 - 7.23 (m, 4H), 7.07 - 7.01 (m, 2H) ppm.

The NMR spectrum is in accordance with the literature values.[S3]

Synthesis of Thiotelluranthrene Te5

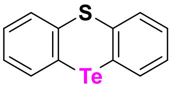

Similar to a known procedure,[S5] a vial was charged with $Pd(OAc)_2$ (56 mg, 0.25 mmol, 5 mol %), DPEphos (270 mg, 0.5 mmol, 10 mol %) and NaO*t*-Bu (961 mg, 10 mmol, 2.0 equiv), was quickly evacuated and flushed with Ar three times. 1-Bromo-2-iodobenzene (0.77 mL, 6.0 mmol, 1.2 equiv), 2-bromobenzenethiol (0.6 mL, 5.0 mmol, 1.0 equiv) and toluene (10 mL) were added under Ar and the reaction mixture was stirred at 110 °C for 24 h. After cooling down to room temperature, the reaction mixture was then diluted with DCM and washed with H_2O and brine. The combined organic layers were dried over Na_2SO_4 and concentrated in vacuo. The crude reaction mixture was purified *via* column chromatography to yield bis(2-bromophenyl)sulfane as a white solid (1.170 g, 3.4 mmol, 68%). Similar to a known procedure,[S1,S2] a vial was charged with bis(2-bromophenyl)sulfane (524 mg, 1.5 mmol, 1.0 equiv), copper(I) iodide (29 mg, 0.15 mmol, 10 mol%) and sodium iodide (904 mg, 6.0 mmol, 4.0 equiv), evacuated and flushed with Ar three times. *N,N*-Dimethylethylendiamine (DMEDA, 32 µL, 0.3 mmol, 20 mol %) and dry 1,4-dioxane (3 mL) were added under Ar. The reaction was stirred Ar 110 °C for 24 h. After cooling down to room temperature, 25% NH_3 solution was added and the mixture was diluted with H_2O. The aqueous layers were extracted with DCM and the combined organic layers were dried over Na_2SO_4. The solvent was removed in vacuo and the crude oil was purified *via* column chromatography using pure pentane to yield bis(2-iodophenyl)sulfane as a white solid (535 mg, 1.2 mmol, 81%). Thiotelluranthrene **Te5** was synthesized based on the procedure described by Cremer *et al*. for 10*H*-phenotellurarzine.[S1] A vial was charged with bis(2-iodophenyl)sulfane (535 mg, 1.2 mmol, 1.0 equiv), tellurium powder (309 mg, 2.4 mmol, 2.0 equiv) and KOH (269 mg, 4.8 mmol), evacuated and flushed with Ar three times. Dry DMSO (3 mL) were added under Ar and the reaction was stirred at 110 °C for 24 h. After cooling down to room temperature, the reaction mixture was diluted with sat. ammonium chloride solution and brine. The aqueous layers were extracted with DCM, the combined organic layers were dried over Na_2SO_4 and the solvent was removed in vacuo. The crude product was purified *via* column chromatography using pentane/ethyl acetate 100:1 to yield the desired product as yellow crystals (208 mg, 0.67 mmol, 56%).

^{1}H-NMR (400 MHz, $CDCl_3$): δ = 7.84 (dd, *J* = 7.5, 1.4 Hz, 2H), 7.73 (dd, *J* = 7.8, 1.4 Hz, 2H), 7.28 (td, *J* = 7.5, 1.5 Hz, 2H), 7.16 (td, *J* = 7.5, 1.4 Hz, 2H) ppm.

^{13}C-NMR (101 MHz, $CDCl_3$): δ = 139.84, 136.26, 130.59, 128.62, 128.14, 123.37 ppm.

^{125}Te-NMR (126 MHz, $CDCl_3$): δ = 672.69 (s) ppm.

IR (neat, cm^{-1}): 3760, 3692, 3044, 2921, 2852, 2674, 2325, 2109, 1994, 1921, 1887, 1804, 1733, 1621, 1548, 1475, 1423, 1244, 1158, 1108, 1081, 1023, 979, 943, 869, 747.

HRMS (APCI, m/z): calculated for $C_{12}H_9STe^+$ ($[M+H]^+$): 314.94818, found: 314.94802.

m.p. 119 / 123 °C

Synthesis of 10-Telluranthracene-9-one Te6

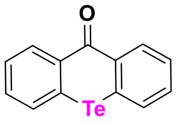

Similar to a known procedure,[S6] a vial was charged with 2-bromophenylboronic acid (603 mg, 3.0 mmol, 1.0 equiv), $Pd(PPh_3)_2Cl_2$ (105 mg, 0.15 mmol, 5 mol%) and K_3PO_4 (1922 mg, 9.0 mmol, 3.0 equiv), evacuated and flushed with Ar three times. 2-Bromobenzoylchloride (0.4 mL, 3.0 mmol, 1.0 equiv) and toluene (10 mL) were added under Ar. The reaction was stirred at 100 °C overnight. After cooling down to room temperature, the pale-yellow reaction mixture was diluted with DCM and extracted with H_2O. The combined organic layers were dried over Na_2SO_4 and the solvent was removed in vacuo. The crude yellow oil was purified *via* column chromatography using pentane/ethyl acetate 50:1 to yield the 2,2'-dibromobenzophenone as a yellow oil (276 mg, 0.81 mmol, 27%). Similar to a known procedure,[S1,S2] a vial was charged with copper(I) iodide (15 mg, 0.08 mmol, 10 mol %) and sodium iodide (482 mg, 3.2 mmol, 4.0 equiv), evacuated and flushed with Ar three times. DMEDA (17 µL, 0.16 mmol, 20 mol %), 2,2'-dibromobenzophenone (276 mg, 0.81 mmol, 1.0 equiv) and dry 1,4-dioxane (3 mL) were added under Ar. The reaction mixture was stirred at 110 °C for 24 h. After cooling down to room temperature, 25% NH_3 solution was added and the mixture was diluted with H_2O. The aqueous layers were extracted with DCM and the combined organic layers were dried over Na_2SO_4. The solvent was removed in vacuo and the crude oil was purified *via* column chromatography using pentane/ethyl acetate to yield 2,2'-diiodobenzophenone as a yellow solid (261 mg, 0.6 mmol, 75%).10-Telluranthracene-9-one **Te6** was synthesized based on the procedure described by Cremer et al. for 10*H*-phenotellurarzine.[S1] A vial was charged with 2,2'-diiodobenzophenone (253 mg, 0.6 mmol, 1.0 equiv), tellurium powder (154 mg, 1.2 mmol, 2.0 equiv) and KOH (140 mg, 2.4 mmol, 4.0 equiv), evacuated and flushed with Ar three times. Dry DMSO (2 mL) was added under Ar and the reaction was stirred at 110 °C for 24 h. After cooling down to room temperature, the reaction mixture was diluted with sat. ammonium chloride solution and brine. The aqueous layers were extracted with DCM, the combined organic layers were dried over Na_2SO_4 and the solvent was removed on silica. The crude product was purified via column chromatography using pentane/ethyl acetate 10:1 to yield the desired product as yellow solid (25 mg, 0.08 mmol, 13%). Note: small impurities were detected in the NMR spectra.

^{1}H-NMR (600 MHz, $CDCl_3$): δ = 8.67 - 8.60 (m, 2H), 7.72 - 7.68 (m, 2H), 7.45 - 7.36 (m, 4H) ppm.

^{13}C-NMR (151 MHz, $CDCl_3$): δ = 186.62, 134.71, 133.94, 133.12, 132.02, 127.85, 120.11 ppm.

^{125}Te-NMR (126 MHz, $CDCl_3$): δ = 468.66 (t, J = 27.3 Hz) ppm. (Literature: 473.4 ppm)[S14]

The NMR data are in accordance with the literature values.[S14]

Synthesis of 9-Phenyl-9*H*-telluroxanthrene Te7

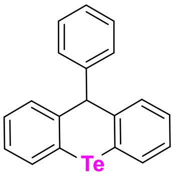

Similar to known procedures,[S7,S15] 1-iodo-2-bromobenzene (0.76 mL, 5.9 mmol, 1.18 equiv) was degassed and cooled down to −15 °C. Isopropylmagnesium chloride (2 M in THF, 3.0 mL, 6 mmol, 1.2 equiv) was added dropwise in 5 minutes under Ar. The reaction mixture was stirred at -15 °C for 2 h. The mixture was then cooled down to −25 °C and 2-bromobenzaldehyde (0.58 mL, 5.0 mmol, 1.0 equiv) was added dropwise over 5 minutes. The reaction mixture was stirred at −25 °C for 75 minutes. The cooling source was removed and the reaction mixture was stirred at room temperature overnight. The reaction was quenched by the addition of sat. ammonium chloride solution and water. Et_2O was added for dilution. The aqueous layers were extracted with Et_2O and the combined organic layers were dried over $MgSO_4$. The solvent was removed and the bis(2-bromophenyl)methanol was obtained as colorless solid (1133 mg, 3.3 mmol, 66%). Similar to a known procedure,[S7] bis(2-bromophenyl)methanol (85 mg, 2.5 mmol, 1.0 equiv) was weighed into a vial. Benzene (1.11 mL, 12.5 mmol, 5.0 equiv), 1,1,1-3,3,3-hexafluoro*iso*propanol (HFIP, 2.5 mL) and triflic acid (22 µL, 0.25 mmol, 10 mol%) were added and the vial was sealed with an aluminous headspace cap. The reaction was stirred at 60 °C for 16 h. After cooling down to room temperature, the solvent was removed and methanol was added to precipitate a yellow solid. The solvent was removed on silica and the crude reaction mixture was purified via column chromatography using pure hexane to yield 2,2'-dibromotriphenylmethane as a colorless solid (762 mg, 1.89 mmol, 76%). 2,2'-Diiodotriphenylmethane was synthesized using the conditions of the aromatic Finkelstein reaction as described by Cremer et al.[S1,S2] A vial was charged with 2,2'-dibromotriphenylmethane (604 mg, 1.5 mmol, 1.0 equiv), copper(I) iodide (30 mg, 0.15 mmol, 10 mol%) and sodium iodide (905 mg, 6.0 mmol, 4.0 equiv), evacuated and flushed with Ar three times. DMEDA (32 µL, 0.3 mmol, 20 mol%) and dry 1,4-dioxane (3.5 mL) were added under Ar and the reaction mixture was stirred at 110 °C for 48 h. After cooling down to room temperature, saturated ammonium chloride solution was added. The aqueous phase was extracted with DCM and the combined organic layers were washed with brine, saturated ammonium chloride solution and dried over Na_2SO_4. The solvent was removed and a crude NMR was measured. The product was thus found to be only partly iodinated. Therefore, the reaction was performed again. The previous 2,2'-(phenylmethylene)bis(bromo/iodobenzene) mixture, copper(I) iodide (41 mg, 0.21 mmol) and sodium iodide (1108 mg, 7.35 mmol) were weighed into a vial. The vial was sealed with an aluminous headspace cap and evacuated and flushed with Ar three times. DMEDA (46 µL, 0.42 mmol) and dry 1,4-dioxane (3.0 mL) was added under Ar and the reaction mixture was stirred at 110 °C for 72 h. After cooling down to room temperature, saturated ammonium chloride solution was added. The aqueous phase was extracted with DCM and the combined organic layers were washed with brine, saturated ammonium chloride solution and dried over Na_2SO_4. The solvent was removed and the crude reaction mixture was purified via column chromatography using pure pentane to yield a 2,2'-(phenylmethylene)bis(bromo/iodobenzene) mixture (Br:I ratio estimated via ^{1}H NMR at 62:38, colorless solid). A vial was charged with this substance mixture, tellurium powder (301 mg, 2.36 mmol) and KOH (276 mg), evacuated and flushed with Ar three times. Dry DMSO (3 mL) was added under Ar and the reaction mixture was stirred at 150 °C for 24 h. After cooling down to room temperature, sat. ammonium chloride solution was added. The aqueous layers were extracted with DCM and the combined

organic layers were washed with brine, sat. ammonium chloride solution and dried over Na_2SO_4. The solvent was removed on silica and the crude product was purified *via* column chromatography using hexane/DCM 3:1 to yield 9-phenyl-9*H*-telluroxanthrene **Te7** as yellow solid (243 mg, 0.66 mmol, 44%).

^{1}H-NMR (400 MHz, $CDCl_3$): δ =7.77 (dd, *J* = 7.5, 1.3 Hz, 2H), 7.55 (d, *J* = 7.7 Hz, 2H), 7.34 (td, *J* = 7.5, 1.3 Hz, 2H), 7.20 - 7.08 (m, 5H), 6.87 - 6.74 (m, 2H), 5.54 (s, 1H) ppm.

^{13}C-NMR (101 MHz, $CDCl_3$): δ = 142.09, 136.36, 131.51, 128.51, 127.81, 127.80, 127.16, 126.29, 117.59, 61.38 ppm.

^{125}Te-NMR (126 MHz, $CDCl_3$): δ = 549.7 - 549.4 (m) ppm.

IR (neat, cm^{-1}): 3393, 3053, 2996, 2922, 2326, 2111, 1920, 1803, 1702, 1574, 1488, 1434, 1340, 1275, 1232, 1184, 1109, 1076, 1024, 977, 945, 915, 843, 728, 696.

HRMS (APCI, m/z) calculated for $C_{19}H_{15}Te^+$ ($[M+H]^+$): 373.02306, found: 373.02217.

m. p. 138 / 142 °C.

Synthesis of 2,8-dimethoxy-10*H*-phenotellurazine PTeZ15

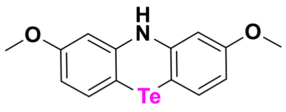

Similar to a known procedure,[S16] 2-bromo-5-methoxyaniline (1.3 mL, 10.0 mmol, 1.0 equiv) was dissolved in MeCN (30 mL) at room temperature. *p*TSA(H_2O) (5708 mg, 30.0 mmol, 3.0 equiv) was added and the mixture was stirred for 10 min. The mixture was cooled down to 0 °C. $NaNO_2$ (1382 mg, 20.0 mmol, 2.0 equiv) in H_2O was added dropwise followed by the dropwise addition of sodium iodide (3756 mg, 25.0 mmol, 2.5 equiv) in H_2O resulting in orange foam. The mixture was stirred at 0 °C for 10 min and then 3 h at room temperature. H_2O (50 mL) was added and $NaHCO_3$ solution (1 M) until reaching pH 9. $Na_2S_2O_3$ (2 M, 6 mL) were added and the aqueous layers were extracted with EtOAc. The combined organic layers were dried over $MgSO_4$ and the solvent was removed. The crude product was purified *via* column chromatography to yield 3-iodo-4-bromoanisole as a yellow oil (2420 mg, 7.7 mmol, 77%).[S17] Similar to a known procedure,[S1,S2] 4-bromo-3-iodoanisole (1921 mg, 6.1 mmol, 1.2 equiv), 2-bromo-5-methoxyaniline (0.67 mL, 5.1 mmol, 1.0 equiv), NaO*t*-Bu (686 mg, 7.14 mmol, 1.4 equiv), $Pd_2(dba)_3$, (238 mg, 0.26 mmol, 5 mol %) and DPPF (286 mg, 0.51 mmol, 10 mol%) were weighed into a vial. Toluene (15 mL) was added and the vial was sealed with an aluminous headspace cap. The reaction mixture was stirred at 120 °C for 19.5 h. After cooling down to room temperature, sat. ammonium chloride was added and the aqueous layer was extracted with EtOAc and the combined organic layers were dried over Na_2SO_4. The solvent was removed on silica and the crude product was purified *via* column chromatography to yield bis(2-bromo-5-methoxyphenyl)amine as clear yellow oil that turned solid at the light (1451 mg, 3.75 mmol, 74%). Similar to a known procedure,[S1,S2] a vial was charged with bis(2-bromo-5-methoxyphenyl)amine (1451 mg, 3.8 mmol, 1.0 equiv), copper(I) iodide (72 mg, 0.38 mmol, 10 mol %) and sodium iodide (2255 mg, 15 mmol, 4.0 equiv), evacuated and flushed with Ar three times. DMEDA (80 µL, 0.75 mmol, 20 mol %) and dry 1,4-dioxane (8 mL) were added under Ar. The reaction mixture was stirred at 110 °C for 22 h. After cooling down to room temperature, 25% NH_3 solution was added and the mixture was diluted with H_2O. The aqueous

layers were extracted with DCM and the combined organic layers were dried over Na_2SO_4. The solvent was removed in vacuo and the crude product was purified *via* column chromatography using hexane/DCM 6:4 to yield bis(2-iodo-5-methoxyphenyl)amine as colorless fluffy solid (1544 mg, 3.2 mmol, 84%). 2,8-Dimethoxy-10*H*-phenotellurazine **PTeZ15** was synthesized based on the procedure described by Cremer et al. for 10*H*-phenotellurarzine.[S1] A vial was charged with bis(2-iodo-5-methoxyphenyll)amine (1000 mg, 2.08 mmol, 1.0 equiv), tellurium powder (529 mg, 4.16 mmol, 2.0 equiv) and KOH (465 mg, 8.32 mmol, 4.0 equiv), evacuated and flushed with Ar three times. Dry DMSO (8 mL) was added under Ar and the reaction was stirred at 110 °C for 24 h. After cooling down to room temperature, the reaction mixture was diluted with sat. ammonium chloride solution and brine. The aqueous layers were extracted with DCM, the combined organic layers were dried over Na_2SO_4 and the solvent was removed on silica. The crude product was purified via column chromatography using hexane/DCM 1:2 to yield the 2,8-Dimethoxy-10*H*-phenotellurazine as a brown solid (280 mg, 0.79 mmol, 38%).

^{1}H-NMR (600 MHz, $CDCl_3$): δ = 7.24 (d, *J* = 7.7 Hz, 2H), 6.49 (dd, *J* = 7.8, 2.3 Hz, 2H), 6.36 (broad s, 2H), 6.10 (broad s, 1H), 3.77 (s, 6H) ppm.

^{13}C-NMR (151 MHz, $CDCl_3$): δ = 160.86, 145.70, 135.52, 109.76, 103.11, 87.15, 55.49 ppm.

^{125}Te-NMR (190 MHz, $CDCl_3$): δ = 396.0 – 395.7 (m) ppm.

IR (neat, cm^{-1}): 3888, 3631, 3331, 3185, 3115, 3047, 3004, 2940, 2840, 2764, 2661, 2570, 2495, 2331, 2232, 2188, 2166, 2116, 1995, 1951, 1918, 1881, 1801, 1742, 1652, 1571, 1491, 1436, 1392, 1313, 1278, 1245, 1203, 1169, 1109, 1045, 1021, 981, 915, 828, 802, 742, 685.

HRMS (ESI, m/z) calculated for $C_{14}H_{14}NO_2Te^+$ ($[M+H]^+$): 358.00813, found 358.00776.

m. p. 174 / 204 °C (decomposition).

Synthesis of *N,N*-dimethyl-10*H*-phenotellurazinediamine (PTeZ16)

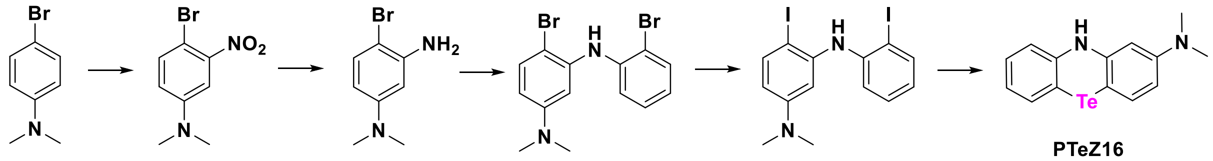

4-Bromo-*N*,*N*-dimethylamino-3-nitroaniline was synthesized in accordance with the procedure presented by Zilate et al.[S8] 4-Bromo-*N*,*N*-dimethylaniline (15005 mg, 75 mmol, 1.0 equiv) was dissolved in Et_2O/THF 1:1 (102 mL). HNO_3 (62%, 75 mmol, 1.0 equiv, 5.4 mL) was added dropwise. The reaction mixture was stirred at room temperature for 1 h. The colorless precipitate was filtered off and washed with Et_2O. The pale-yellow salt was dried overnight. The salt was dissolved in DCM (54 mL). At 0 °C, H_2SO_4 (95-97 %, 16.5 mL) was added dropwise. The ice bath was removed and the mixture was stirred at rt for 1 h. The reaction mixture was slowly added to cold water (54 mL). 25% NH_3 was added until pH = 10. The aqueous layers were extracted with DCM and the combined organic layers were dried over $MgSO_4$ and the solvent was removed to obtain 4-bromo-*N*,*N*-dimethylamino-3-nitroaniline as an orange solid (7833 mg, 32.0 mmol, 43%). 4-Bromo-N^1,N^1-dimethylbenzene-1,3-diamine was synthesized in accordance with the procedure presented by Fischer and Sparr.[S9] 4-

Bromo-*N*,*N*-dimethylamino-3-nitroaniline (9802 mg, 40.0 mmol, 1.0 equiv) was stirred in 32% HCl at 0 °C. $SnCl_2(H_2O)_2$ (27088 mg, 120 mmol, 3.0 equiv) was slowly added. The ice bath was removed and the reaction mixture was stirred at room temperature for 24 h. The, the reaction mixture was poured on ice and 25% NH_3 was added until pH 10. The mixture was washed with H_2O and DCM. The aqueous phase was extracted with DCM and the combined organic layers were dried over $MgSO_4$. The crude product was purified by filtration over a silica pad using Hex/DCM 1:1 to pure DCM to yield 4-bromo-N^1,N^1-dimethylbenzene-1,3-diamine as a beige solid (6116 mg, 28.4 mmol, 71%). 4-Bromo-N^3-(2-bromophenyl)-N^1,N^1-dimethylbenzene-1,3-diamine was synthesized in accordance with the procedure presented by Cremer et al.[S1,S2] 4-Bromo-N^1,N^1-dimethylbenzene-1,3-diamine (1076 mg, 5.0 mmol, 1.0 equiv), 1-bromo-2-iodobenzene (0.77 mL, 6.0 mmol, 1.2 equiv), NaO*t*-Bu (673 mg, 7.0 mmol, 1.4 equiv), $Pd_2(dba)_3$, (226 mg, 0.25 mmol, 5 mol%) and DPPF (281 mg, 0.5 mmol, 10 mol%) were weighed into a vial. Toluene (11 mL) was added and the vial was sealed with an aluminous headspace cap. The reaction mixture was stirred at 120 °C for 18 h. After cooling down to room temperature, sat. ammonium chloride was added and the aqueous layer was extracted with EtOAc and the combined organic layers were dried over $MgSO_4$. The solvent was removed on silica and the crude product was purified *via* column chromatography hexane/DCM 6:4 to yield 4-bromo-N^3-(2-bromophenyl)-N^1,N^1-dimethylbenzene-1,3-diamine as an orange solid (1108 mg, 2.99 mmol, 60%). 4-Iodo-N^3-(2-iodoophenyl)-N^1,N^1-dimethylbenzene-1,3-diamine was synthesized in accordance with the procedure for the aromatic Finkelstein reaction presented by Cremer *et al.*[S1,S2] A vial was charged with copper(I) iodide (49 mg, 0.25 mmol, 10 mol%) and sodium iodide (1536 mg, 10.2 mmol, 4.1 equiv), evacuated and flushed with Ar three times. DMEDA (54 µL, 0.5 mmol, 20 mol %), 4-bromo-N^3-(2-bromophenyl)-N^1,N^1-dimethylbenzene-1,3-diamine (927 mg, 2.5 mmol, 1.0 equiv) in dry 1,4-dioxane (8 mL) were added under Ar. The reaction mixture was stirred at 110 °C for 24 h. After cooling down to room temperature, 25% NH_3 solution was added and the mixture was diluted with H_2O. The aqueous layers were extracted with DCM and the combined organic layers were dried over $MgSO_4$. The solvent was removed *in vacuo* and the crude product was purified *via* column chromatography using hexane/DCM to yield 4-Iodo-N^3-(2-iodoophenyl)-N^1,N^1-dimethylbenzene-1,3-diamine as colorless crystals (1005 mg, 2.2 mmol, 85%). *N,N*-Dimethyl-10H-phenotellurazin-2-amine **PTeZ16** was synthesized based on the procedure described by Cremer *et al*. for 10*H*-phenotellurarzine.[S1] A vial was charged with 4-iodo-N^3-(2-iodoophenyl)-N^1,N^1-dimethylbenzene-1,3-diamine (928 mg, 2.0 mmol, 1.0 equiv), tellurium powder (511 mg, 4.0 mmol, 2.0 equiv) and KOH (452 mg, 8.0 mmol, 4.0 equiv), was evacuated and flushed with Ar three times. Dry DMSO (8 mL) was added under Ar and the reaction mixture was stirred at 120 °C for 24 h. After cooling down to room temperature, the reaction mixture was diluted with sat. ammonium chloride solution and brine. The aqueous layer was extracted with DCM, the combined organic layers were dried over $MgSO_4$ and the solvent was removed in vacuo. The crude product was purified *via* column chromatography using hexane/ethyl acetate 2:1 to yield the *N,N*-dimethyl-10H-phenotellurazin-2-amine **PTeZ16** as an orange solid (333 mg, 1.0 mmol, 50%).

^{1}H-NMR (600 MHz, $CDCl_3$): δ = 7.38 (d, *J* = 7.5 Hz, 1H), 7.17 (d, *J* = 8.4 Hz, 1H), 7.07 (td, *J* = 7.6, 1.4 Hz, 1H), 6.83 (td, *J* = 7.4, 1.2 Hz, 1H), 6.73 (d, *J* = 7.9 Hz, 1H), 6.36 (dd, *J* = 8.5, 2.5 Hz, 1H), 6.19 (d, *J* = 2.5 Hz, 1H), 6.13 (d, 1H), 2.92 (s, 6H) ppm.

^{13}C-NMR (151 MHz, $CDCl_3$): δ = 151.72, 145.57, 145.01, 135.25, 135.15, 128.35, 123.38, 116.36, 109.43, 101.21, 98.62, 81.28, 40.65 ppm.

^{125}**Te-NMR** (190 MHz, $CDCl_3$): δ = 390.20 (s) ppm.

IR (neat, cm^{-1}): 3872, 3343, 3160, 3045, 2844, 2795, 2327, 2084, 1989, 1852, 1731, 1579, 1519, 1445, 1347, 1300, 1234, 1158, 1121, 1034, 980, 924, 821, 779, 738, 704.

HRMS (ESI, m/z) calculated for $C_{14}H_{14}N_2Te$ (M^+): 340.02137, found 340.02069.

m. p. 130 / 171 °C (decomposition).

Synthesis of diphenyl tellurides (Te8–13)

General Method

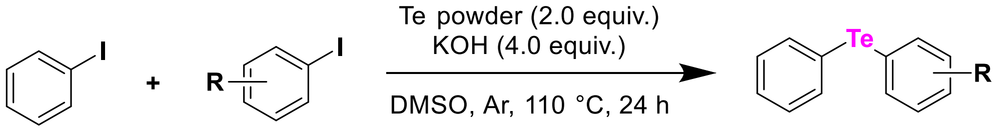

Tellurium powder (5.0 mmol, 2.0 equiv), KOH (10.0 mmol, 4.0 equiv) and any further solid substrate (2.5 mmol, 1.0 equiv) were weighed into a 50 mL vial. The vial was sealed with an aluminous headspace cap, evacuated and flushed with Ar three times. Iodobenzene (0.28 mL, 2.5 mmol, 1.0 equiv), further liquid substrates (2.5 mmol, 1.0 equiv) and dry DMSO (12 mL) were added under Ar. The reaction was stirred at 110 °C for 24 h. After cooling down to room temperature, the reaction mixture was diluted with saturated ammonium chloride solution and DCM. The aqueous phase was extracted with DCM. The combined organic layers were dried over Na_2SO_4. The crude product was purified *via* column chromatography.

Diphenyl telluride Te8

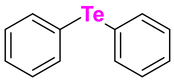

Diphenyl telluride **Te8** was synthesized as a mixture of the methods presented by Cremer et al.[S1] and Jin and co-workers[S18] from tellurium powder (639 mg, 5.0 mmol, 1.0 equiv), KOH (576 mg, 10 mmol, 2.0 equiv) and iodobenzene (0.56 mL, 5.0 mmol, 1.0 equiv). After the aqueous workup, the crude product was purified *via* column chromatography using hexane/DCM in a 1:1 ratio to yield the title compound as orange oil (1111 mg, 3.9 mmol, 79%).

^{1}**H-NMR** (400 MHz, $CDCl_3$): δ = 7.73 (d, J = 7.0 Hz, 2H), 7.34-7.23 (m, 3H) ppm.

^{125}**Te-NMR** (126 MHz, $CDCl_3$): δ = 689.82 (s) ppm.

The NMR spectrum is in accordance with the reported literature.[S19]

(4-Methoxyphenyl)phenyltellane Te9

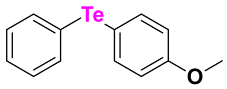

From 4-iodoanilsole (585 mg, 2.5 mmol, 1.0 equiv), iodobenzene (0.28 mL, 2.5 mmol, 1.0 equiv), tellurium powder (641 mg, 5.0 mmol, 2.0 equiv) and KOH (576 mg, 10.0 mmol, 4.0 equiv).
After the aqueous workup, the crude product was purified via column chromatography using hexane/DCM in a 6:4 ratio to yield the title compound as orange oil (347 mg, 1.1 mmol, 44%).

^{1}H-NMR (600 MHz, $CDCl_3$): δ = 7.77 - 7.70 (m, 2H), 7.59 - 7.54 (m, 2H), 7.25 - 7.13 (m, 3H), 6.83 - 6.78 (m, 2H), 3.81 (s, 3H) ppm.

The NMR spectrum is in accordance with the reported literature.[S19]

^{125}Te-NMR (126 MHz, $CDCl_3$): δ = 664.91 (s) ppm.

***N,N*-Dimethyl-4-(phenyltellanyl)aniline Te10**

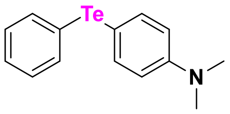

4-Iodo-*N,N*-dimethylaniline was first synthesized according to the procedure presented by Blart, Odobel and co-workers.[S10] *N,N*-Dimethylaniline (0.38 mL, 3.0 mmol, 1.0 equiv) was dissolved in pyridine (20 mL) and 1,4-dioxane (20 mL) at 0 °C. Iodine (2284 mg, 9.0 mmol, 3.0 equiv) was added, turning the mixture brown. The reaction was stirred at 0 °C for 1 h. An additional portion of iodine (785 mg, 3.0 mmol, 1.0 equiv) was added, the ice bath was removed and the mixture was stirred at room temperature for an additional hour. A saturated solution of $Na_2S_2O_3$ was added until the brown color disappeared. The aqueous phase was extracted with DCM and the combined organic layers were washed with water, dried over Na_2SO_4 and the solvent was removed in vacuo. The crude product was purified via column chromatography using hexane/DCM in a 6:4 ratio to yield 4-iodo-*N,N*-dimethylaniline as a green solid (370 mg, 1.5 mmol, 50%). ***N,N*-Dimethyl-4-(phenyltellanyl)aniline Te10:** From 4-iodo-*N,N*-dimethylaniline (245 mg, 1.0 mmol, 1.0 equiv), iodobenzene (0.11 mL, 1 mmol, 1.0 equiv), tellurium powder (260 mg, 2.0 mmol, 2.0 equiv) and KOH (236 mg, 4.0 mmol, 4.0 equiv). After the aqueous workup, the crude product was purified *via* column chromatography using hexane/DCM in a 6:4 ratio to yield *N,N*-dimethyl-4-(phenyltellanyl)aniline **Te10** as an orange oil (13 mg, 0.04 mmmol, 4%).

^{1}H-NMR (600 MHz, $CDCl_3$): δ = 7.71 (d, J = 8.2 Hz, 2H), 7.50 (d, J = 7.6 Hz, 2H), 7.22 - 7.10 (m, 3H), 6.61 (d, J = 8.2 Hz, 2H), 2.98 (s, 6H) ppm.

^{13}C-NMR (101 MHz, $CDCl_3$): δ = 150.69, 141.76, 135.59, 129.32, 126.90, 117.09, 113.72, 97.33, 40.29 ppm.

^{125}Te-NMR (126 MHz, $CDCl_3$): δ = 639.91 (≈t, $J \approx$ 8.8 Hz) ppm.

IR (neat, cm^{-1}): 3417, 3056, 2983, 2885, 2804, 2333, 2105, 1994, 1875, 1805, 1743, 1585, 1498, 1439, 1354, 1226, 1194, 1167, 1128, 1065, 1016, 996, 944, 805, 729, 690.

HRMS (ESI, m/z) calculated for $C_{14}H_{15}NTe$ (M^+): 327.02612, found 327.02533.

(2,4-Dimethoxyphenyl)phenyltellane Te11

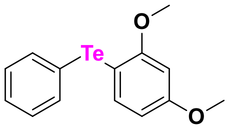

From 1-Iodo-2,4-dimethoxybenzene (660 mg, 2.5 mmol, 1.0 equiv), iodobenzene (0.28 mL, 2.5 mmol, 1.0 equiv), tellurium powder (638 mg, 5.0 mmol, 2.0 equiv) and KOH (563 mg, 10.0 mmol, 4.0 equiv). After the aqueous workup, the crude reaction mixture was purified via column chromatography using hexane/DCM in a 3:1 ratio to yield the title compound as orange oil (233 mg, 0.7 mmol, 27%).

^{1}H-NMR (600 MHz, $CDCl_3$): δ = 7.79 (dd, J = 8.0, 1.3 Hz, 2H), 7.33 (tt, J = 7.4 Hz, J = 1.0 Hz, 1H), 7.27 - 7.22 (m, 2H), 7.08 (d, J = 8.4 Hz, 1H), 6.46 (d, J = 2.4 Hz, 1H), 6.38 (dd, J = 8.4, 2.4 Hz, 1H), 3.84 (s, 3H), 3.78 (s, 3H) ppm.

^{13}C-NMR (151 MHz, $CDCl_3$): δ = 161.57, 159.93, 139.75, 136.43, 129,57, 128.18, 113.26, 106.86, 98.45, 96.10, 56.06, 55.56 ppm.

IR (neat, cm^{-1}): 3146, 3056, 2999, 2936, 2833, 2318, 2081, 1880, 1577, 1458, 1403, 1299, 1252, 1205, 1155, 1055, 1026, 913, 828, 791, 733, 692.

HRMS (APCI, m/z) calculated for $C_{14}H_{14}O_2Te$ (M^+): 344.00505, found 344.00586.

(3,5-Dimethoxyphenyl)phenyltellane Te12

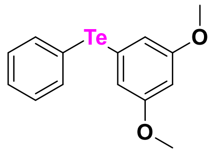

From 3,5-dimethoxyiodobenzene (661 mg, 2.5 mmol, 1.0 equiv), iodobenzene (0.28 mL, 2.5 mmol, 1.0 equiv), tellurium powder (640 mg, 5.0 mmol, 2.0 equiv) and KOH (560 mg, 10.0 mmol, 4.0 equiv). After the aqueous workup, the crude product was purified via column chromatography using hexane/DCM in a 6:4 ratio to yield the title compound as orange oil (111 mg, 0.32 mmol, 13%).

^{1}H-NMR (400 MHz, $CDCl_3$): δ = 7.77 - 7.67 (m, 2H), 7.33 - 7.27 (m, 1H), 7.26 - 7.19 (m, 2H), 6.81 (d, J = 2.3 Hz, 2H), 6.35 (t, J = 2.3 Hz, 1H), 3.73 (s, 6H) ppm.

^{13}C-NMR (101 MHz, $CDCl_3$): δ = 161.13, 138.46, 129.67, 128.16, 115.57, 100.67, 55.50 ppm.

^{125}Te-NMR (126 MHz, $CDCl_3$): δ = 717.36 (t, J = 9.2 Hz) ppm.

IR (neat, cm^{-1}): 3057, 2998, 2936, 2832, 2345, 2195, 2076, 1876, 1809, 1572, 1454, 1414, 1323, 1278, 1199, 1152, 1058, 1037, 990, 922, 829, 731, 686.

HRMS (ESI, m/z) calculated for $C_{14}H_{14}O_2TeNa$ ($[M+Na]^+$): 366.99482, found 366.99388.

(3,4,5-Trimethoxyphenyl)phenyltellane Te13

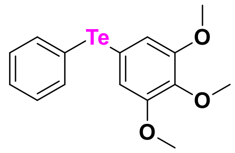

From 3,4,5-trimethoxyiodobenzene (736 mg, 2.5 mmol, 1.0 equiv), iodobenzene (0.28 mL, 2.5 mmol, 1.0 equiv), tellurium powder (641 mg, 5.0 mmol, 2.0 equiv) and KOH (570 mg, 10.0 mmol, 4.0 equiv). After the aqueous workup, the crude reaction mixture was purified via column chromatography using hexane/EtOAc in a 5:1 ratio to yield the title compound as orange oil (233 mg, 0.63 mmol, 25%).

^{1}H-NMR (400 MHz, $CDCl_3$): δ = 7.67 (d, J = 6.8 Hz, 2H), 7.33 - 7.19 (m, 3H), 6.95 (s, 2H), 3.71 (s, 6H), 3.65 (s, 3H) ppm.

^{13}C-NMR (101 MHz, $CDCl_3$): δ = 153.87, 137.45, 129.66, 127.91, 116.12, 115.01, 107.18, 61.07, 56.38 ppm.

^{125}Te-NMR (126 MHz, $CDCl_3$): δ = 723.51 (s) ppm.

HRMS (ESI, m/z) calculated for $C_{15}H_{16}O_3Te$ (M^+): 374.01562, found 374.01462.

IR (neat, cm^{-1}): 3053, 2932, 2829, 2687, 2588, 2469, 2325, 2210, 2084, 1993, 1946, 1810, 1743, 1651, 1571, 1491, 1452, 1396, 1297, 1231, 1176, 1119, 1004, 918, 821, 765, 731, 692.

Catalytic test reactions

The test reactions were performed according to literature procedures.[S1,S2] The results of the test reactions can be found in the results and discussion part of the article.

Test Reaction A:

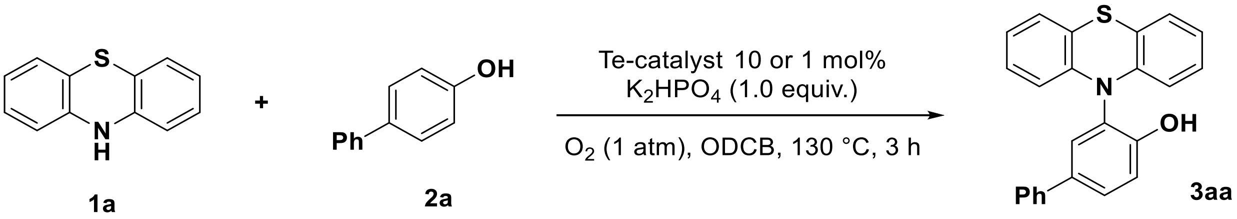

In accordance with the procedure presented by Cremer *et al.*.[S1] 10*H*-Phenothiazine (100 mg, 0.5 mmol, 1.0 equiv), 4-phenylphenol (255 mg, 1.5 mmol, 3.0 equiv), Te-catalyst (0.05 mmol, 10 mol % or 0.005 mmol, 1 mol %) were weighed into a 20 mL vial. ODCB (1.5 mL) was added, the vial was sealed and O_2 (1 atm) was bubbled through for 2 min. The reaction was stirred at 130 °C for 3 h. After cooling down to room temperature, the product was purified via column chromatography using hexane/DCM as solvents in a 6:4 ratio. The product was obtained as colorless solid.

^{1}H-NMR (600 MHz, DMSO-d$_6$): δ = 10.14 (s, 1H), 7.74 (dd, *J* = 8.5, 2.4 Hz, 1H), 7.69 – 7.63 (m, 2H), 7.55 (d, *J* = 2.4 Hz, 1H), 7.40 (dd, *J* = 8.3, 7.3 Hz, 2H), 7.29 (t, *J* = 7.2 Hz, 1H), 7.23 (d, *J* = 8.5 Hz,1H), 7.01 (dd, *J* = 7.6, 1.5 Hz, 2H), 6.9 (ddd, *J* = 8.5, 7.2, 1.6 Hz, 2H), 6.80 (td. *J* = 7.4, 1.3 Hz, 2H), 6.14 (dd, *J* = 8.3, 1.2 Hz, 2H) ppm.

The NMR spectrum is in accordance with the literature values.[S1]

Test Reaction B:

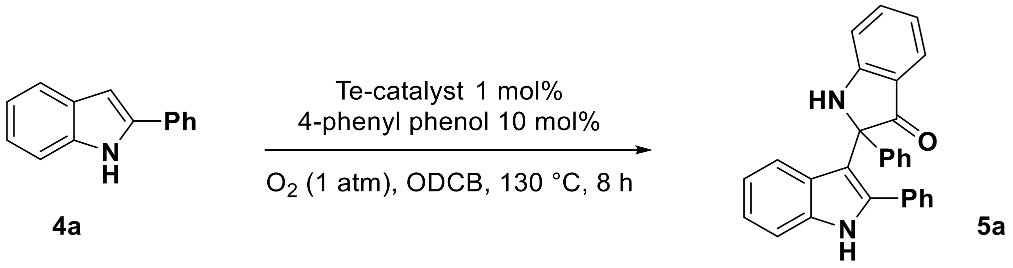

In accordance with the procedure presented by Cremer et al.[S2] 2-Phenylindole (97 mg, 0.5 mmol, 1.0 equiv), Te-catalyst (0.005 mmol, 1 mol %) and 4-phenylphenol (9 mg, 0.05 mmol, 10 mol %) were weighed into a 20 mL vial. ODCB (1.5 mL) was added, the vial

was sealed and O_2 (1 atm) was bubbled through for 2 min. The reaction was stirred at 130 °C for 3 or 8 h. After cooling down to room temperature, the product was purified via column chromatography using hexane/ethyl acetate as solvents in a 8:2 ratio. The product was obtained as yellow oil.

^{1}H-NMR (600 MHz, DMSO-d$_6$): δ = 11.33 (s, 1H), 8.32 (s, 1H), 7.51 (t, *J* = 7.0 Hz, 1H), 7.40 – 7.35 (m, 2H), 7.34 (d, *J* = 8.1 Hz, 1H), 7.25 (d, *J* = 7.7 Hz, 1H), 7.17 – 7.11 (m, 3H), 7.08 – 7.00 (m, 6H), 6.97 (d, *J* = 8.3 Hz, 1H), 6.75 (t, *J* = 7.6 Hz, 1H), 6.71 (t, *J* = 7.4 Hz, 1H), 6.60 (d, *J* = 8.2 Hz, 1H) ppm.

The NMR spectrum is in accordance with the literature values.[S2]

References

[S1] C. Cremer, M. Goswami, C. K. Rank, B. de Bruin, F. W. Patureau, *Angew. Chem. Int. Ed.* **2021**, *60*, 6451.

[S2] C. Cremer, F. W. Patureau, *JACS Au* **2022**, *2*, 1318.

[S3] F. Mostaghimi, E. Lork, I. Hong, T. L. Roemmele, R. T. Boeré, S. Mebs, J. Beckmann, *New J. Chem.* **2019**, *43*, 12754.

[S4] C. Cremer, M. A. Eltester, H. Bourakhouadar, I. L. Atodiresei, F. W. Patureau, *Org. Lett.* **2021**, *23*, 3243.

[S5] L. Zhang, X. Huang, S. Zhen, J. Zhao, H. Li, B. Yuan, G. Yang, *Org. Biomol. Chem.* **2017**, *15*, 6306.

[S6] J. Janke, A. Villinger, P. Ehlers, P. Langer, *Synlett* **2019**, *30*, 817.

[S7] T.-H. Doan, A. Chardon, A. Osi, D. Mahaut, N. Tumanov, J. Wouters, B. Champagne, G. Berionni, *Chem. Eur. J.* **2021**, *27*, 1736.

[S8] B. Zilate, C. Fischer, L. Schneider, C. Sparr, *Synthesis* **2019**, *51*, 4359.

[S9] C. Fischer, C. Sparr, *Angew. Chem. Int. Ed.* **2018**, *57*, 2436.

[S10] C. Monnereau, E. Blart, F. Odobel, *Tetrahedron Lett.* **2005**, *46*, 5421.

[S11] A. V. Kumar, V. P. Reddy, C. S. Reddy, K. R. Rao, *Tetrahedron Lett.* **2011**, *52*, 3978.

[S12] R. E. Humphries, N. A. A. Al-Jabar, D. Bowen, A. G. Massey, G. B. Deacon, *J. Organometallic Chem.* **1987**, *319*, 59.

[S13] C. D. Prasad, S. J. Balkrishna, A. Kumar, B. S. Bhakuni, K. Shrimali, S. Biswas, S. Kumar, *J. Org. Chem.* **2013**, *78*, 1434.

[S14] A. Levy, P. U. Biedermann, S. Cohen, I. Agranat, *J. Chem. Soc., Perkin Trans. 2* **2000**, 725.

[S15] D. Sälinger, R. Brückner, *Chem. Eur. J.* **2009**, *15*, 6688.

[S16] M. Trusova, E. Krasnokutskaya, P. Postnikov, Y. Choi, K.-W. Chi, V. Filimonov, *Synthesis* **2011**, 2154.

[S17] K. S. Madden, B. Laroche, S. David, A. S. Batsanov, D. Thompson, J. P. Knowles, A. Whiting, *Eur. J. Org. Chem.* **2018**, 5312.

[S18] S. Zhang, K. Karra, A. Koe, J. Jin, *Tetrahedron Lett.* **2013**, *54*, 2452.

[S19] N. Sun, K. Zheng, P. Sun, Y. Chen, L. Jin, B. Hu, Z. Shen, X. Hu, *Adv. Synth. Catal.* **2021**, *363*, 3577.

[S20] P. Lorenz, T. Luchs, A. Hirsch, *Chem. Eur. J.* **2021**, *27*, 4993.

[S21] P. Y. Vemuri, C. Cremer, F. W. Patureau, *Org.Lett.* **2022**, *24*, 1626.

NMR Spectra

Se1

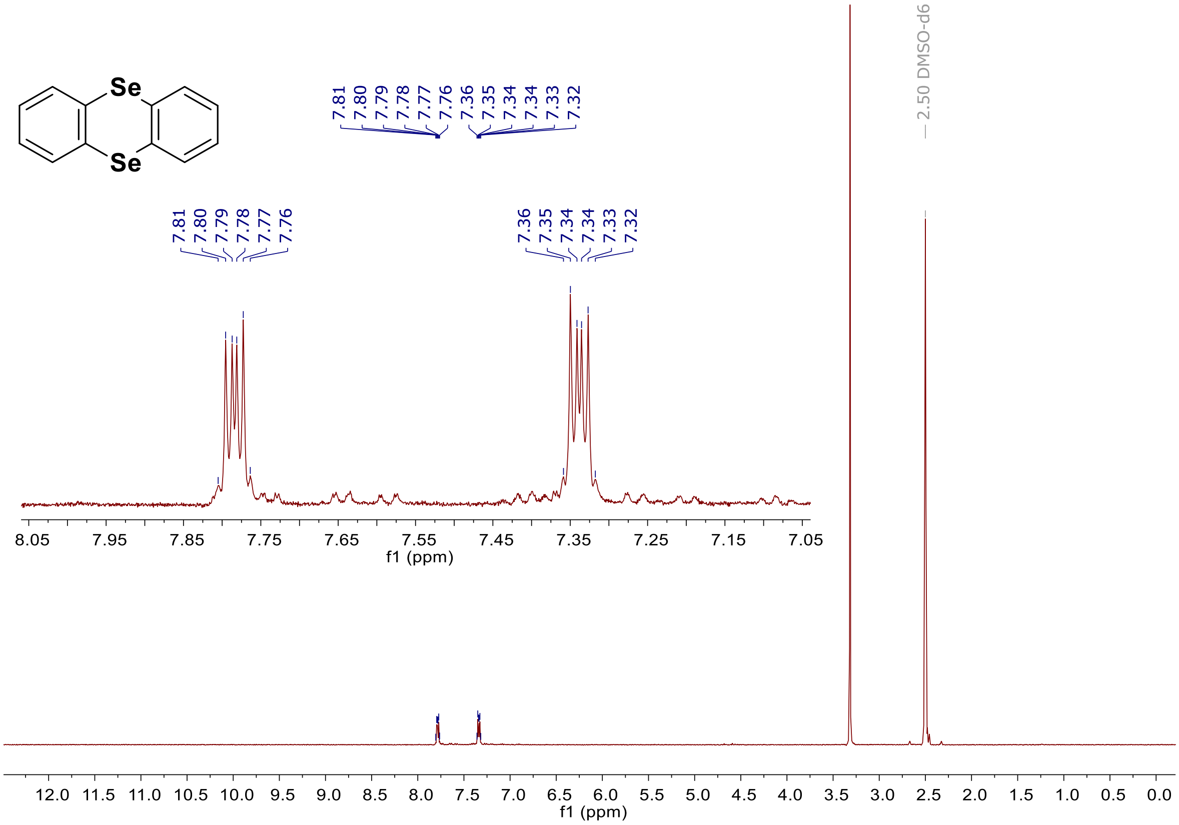

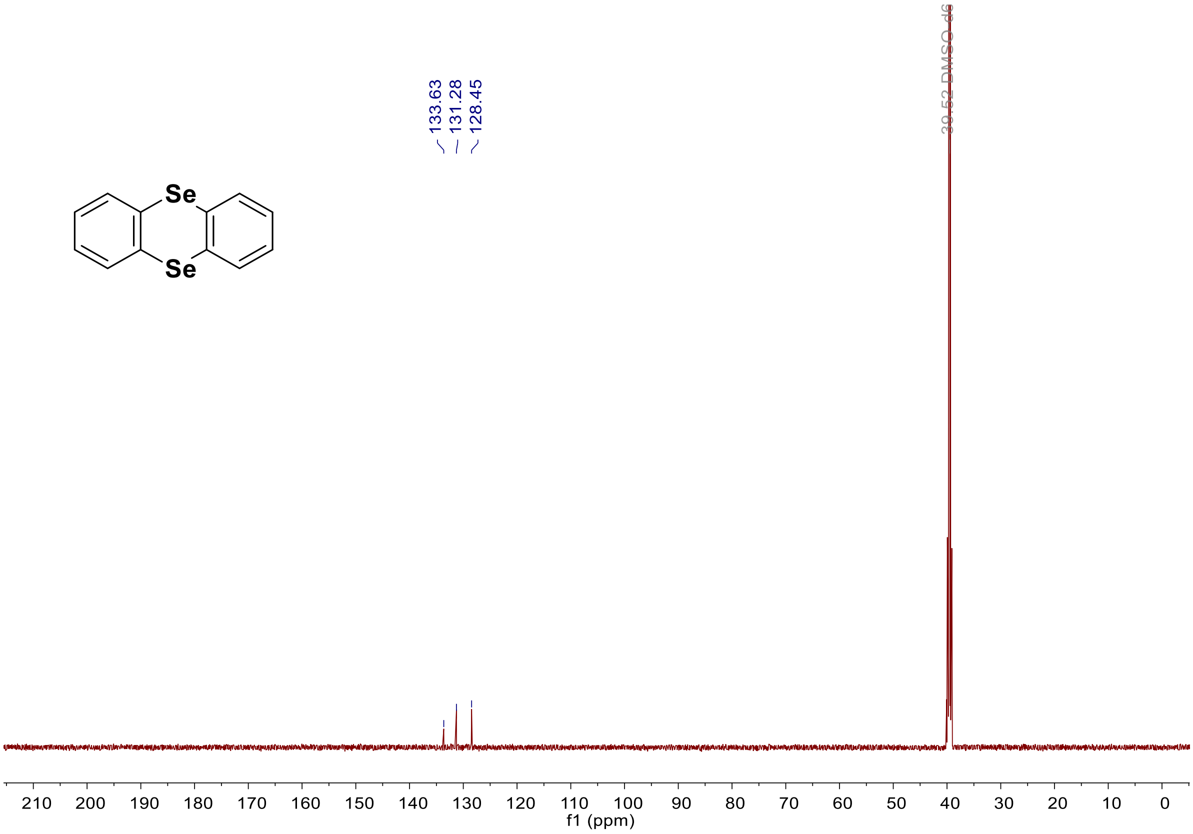

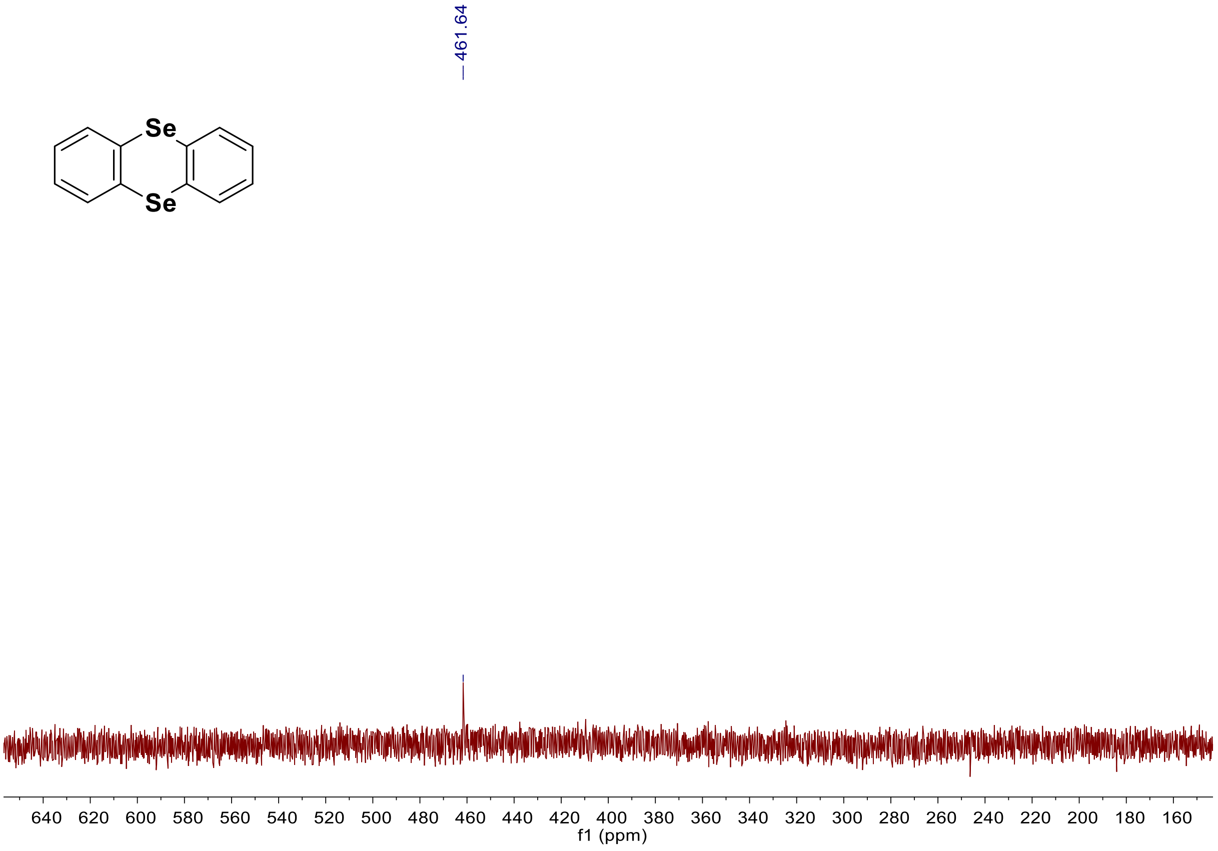

Phenoxatellurine Te4

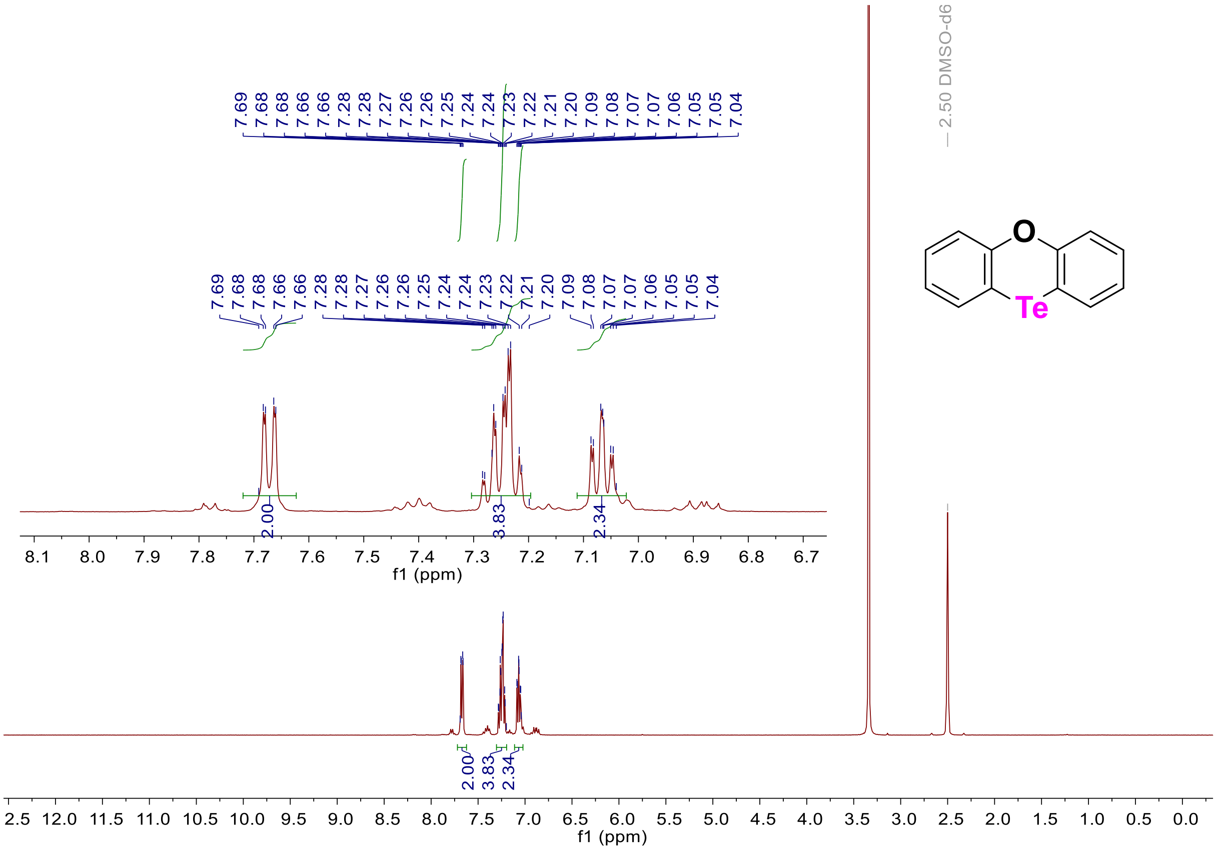

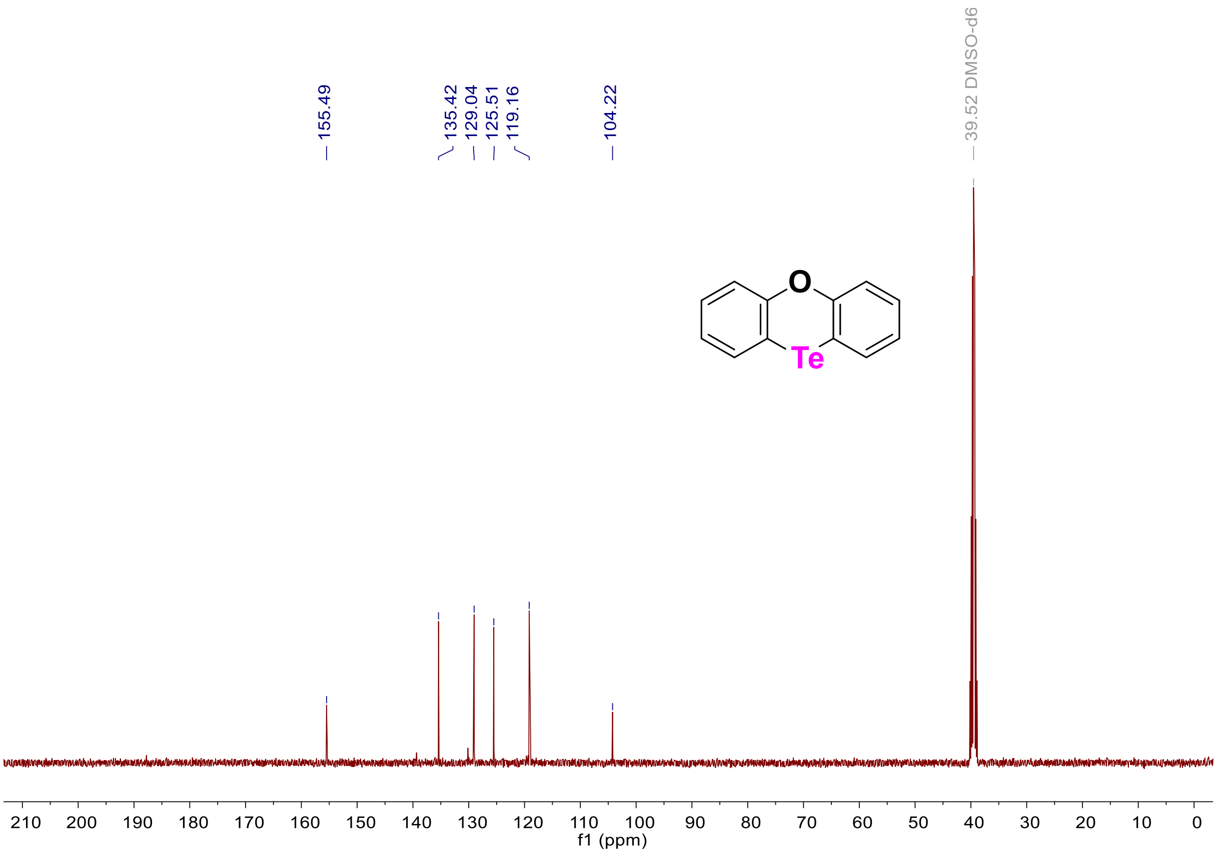
155.49
135.42
129.04
125.51
119.16
104.22
39.52 DMSO-d6
O
Te
210 200 190 180 170 160 150 140 130 120 110 100 90 80 70 60 50 40 30 20 10 0
f1 (ppm)

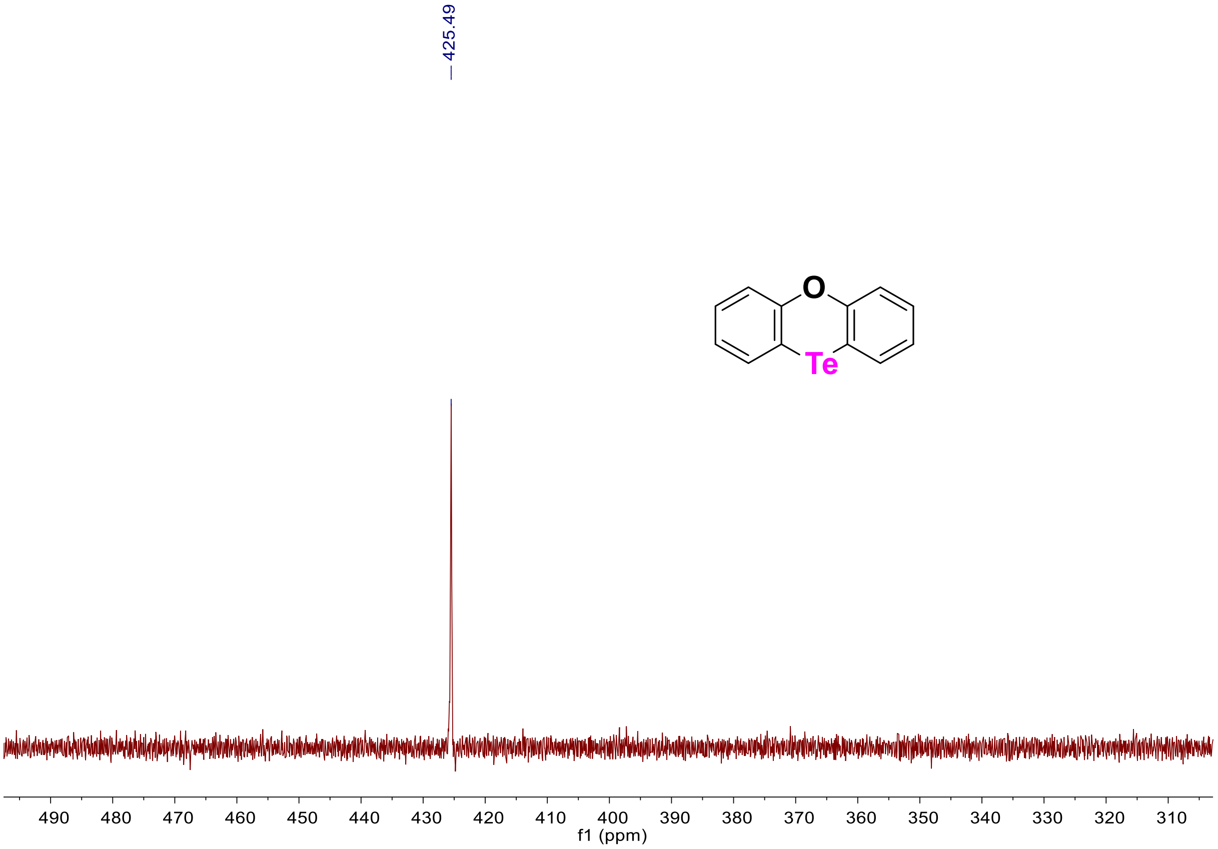
425.49
O
Te
490 480 470 460 450 440 430 420 410 400 390 380 370 360 350 340 330 320 310
f1 (ppm)

Thiotelluranthrene Te5

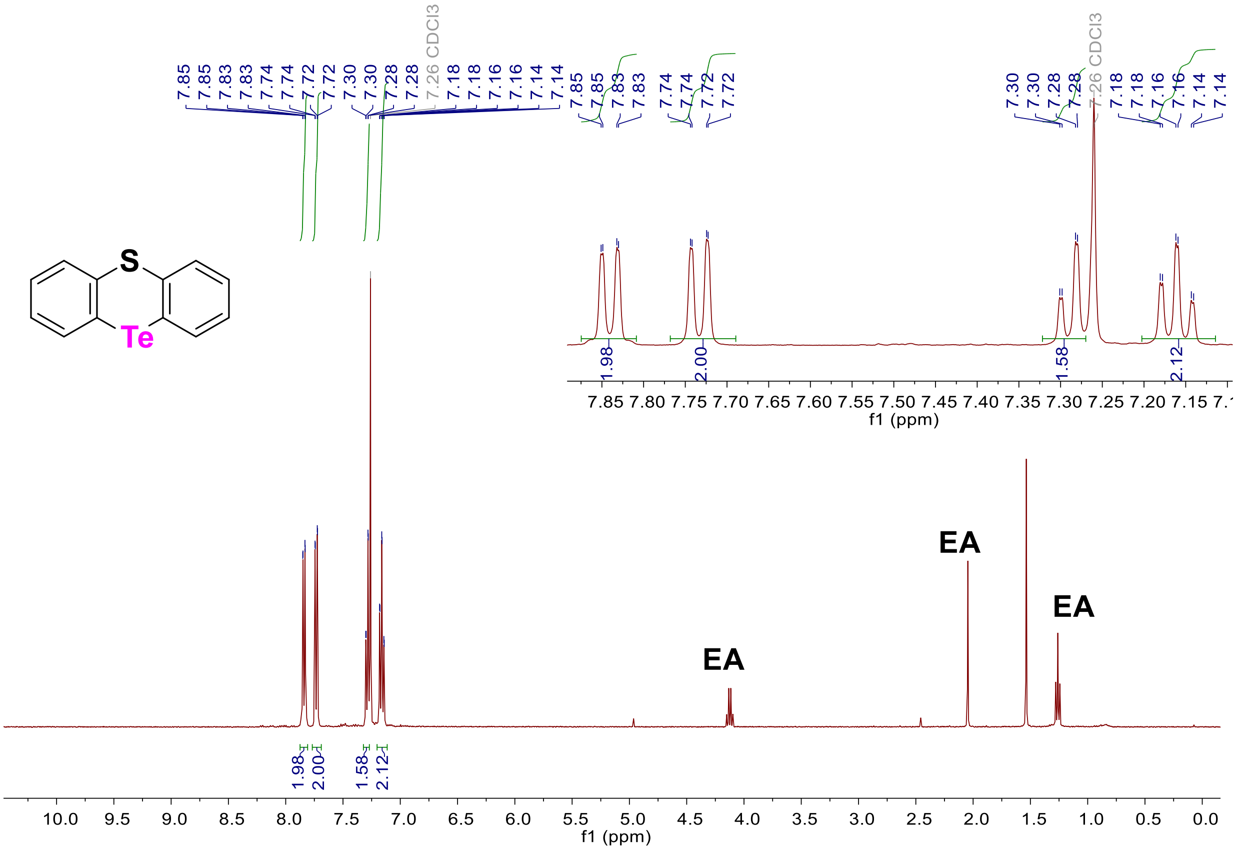

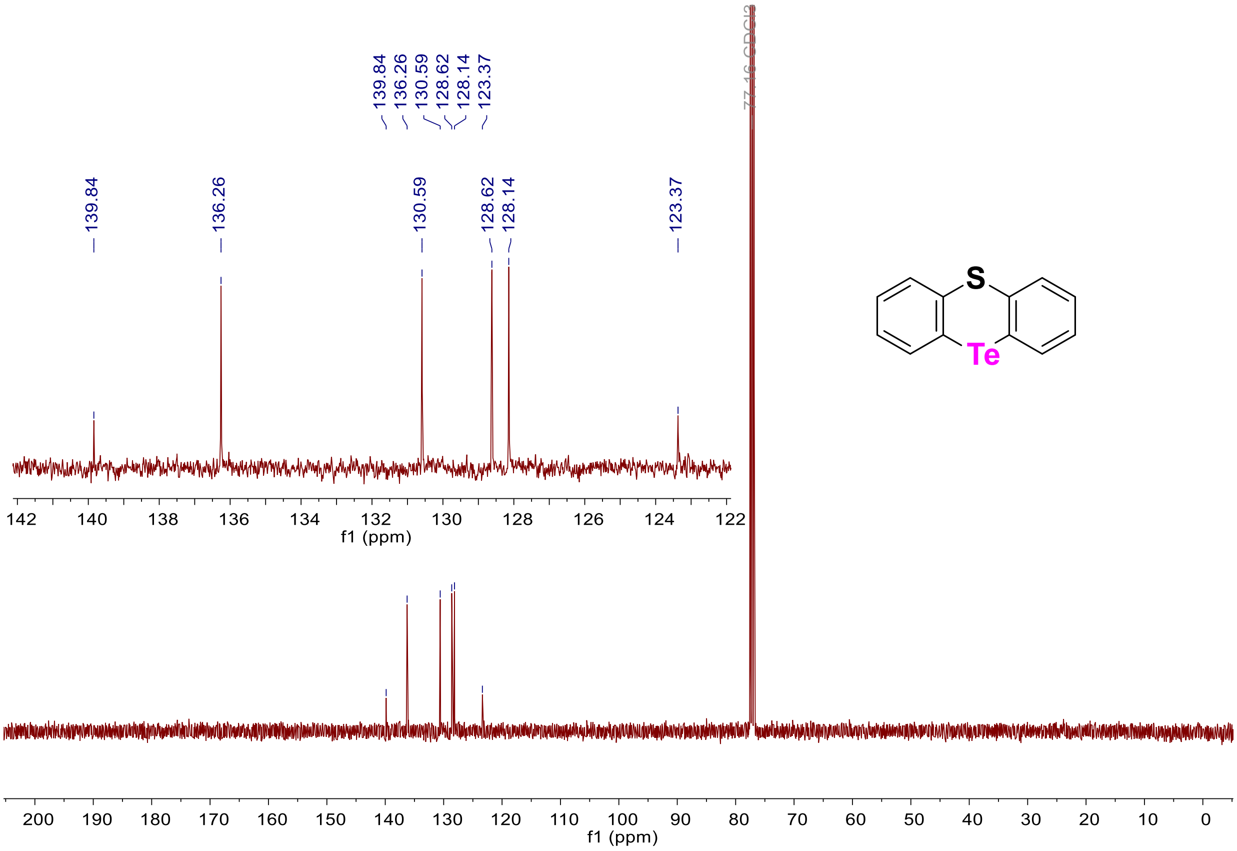

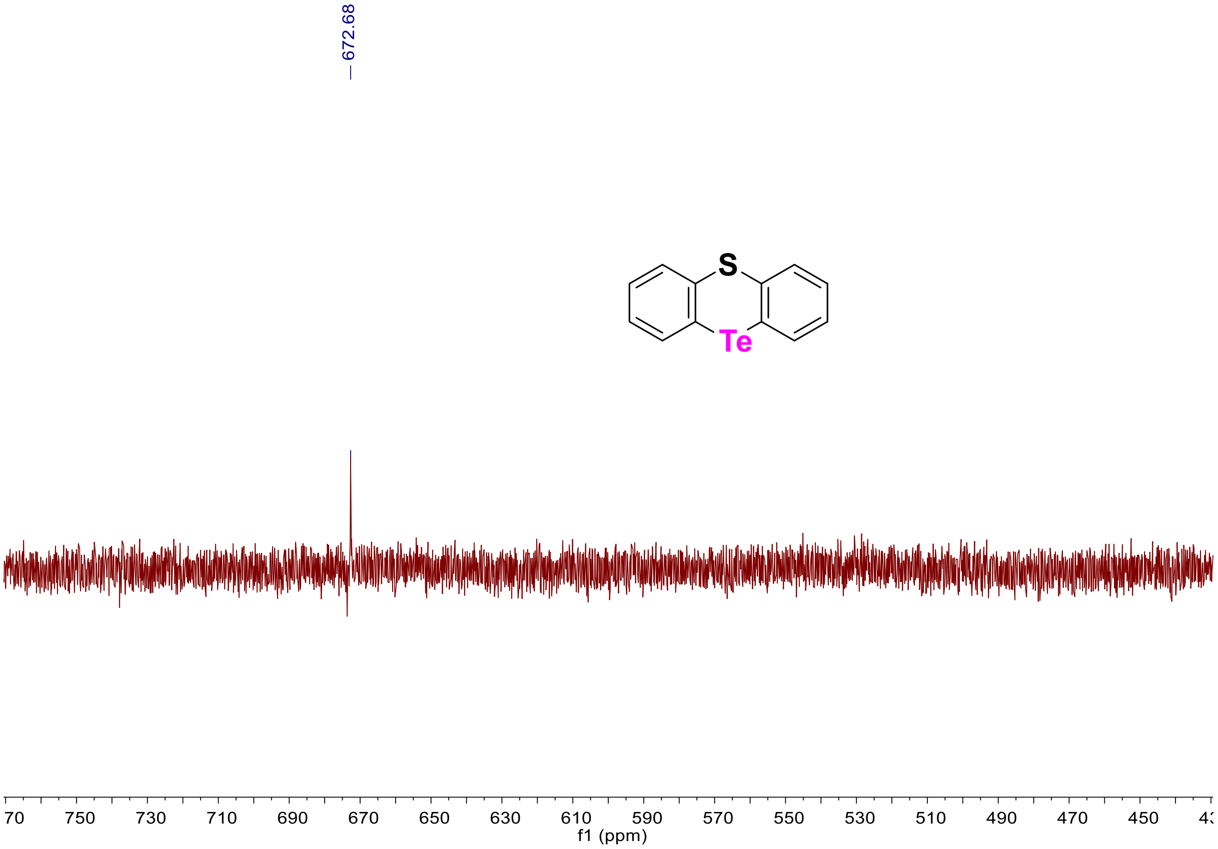

10-Telluranthracene-9-one Te6

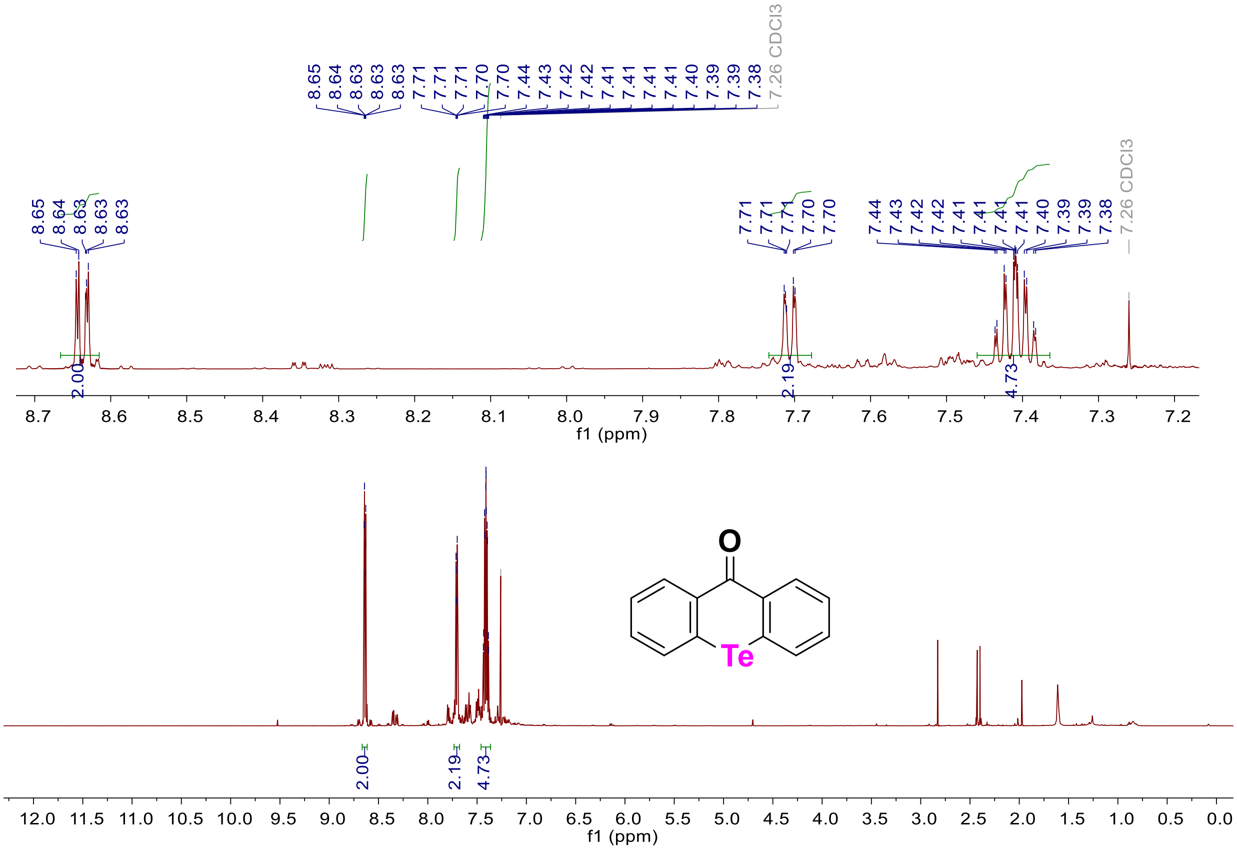

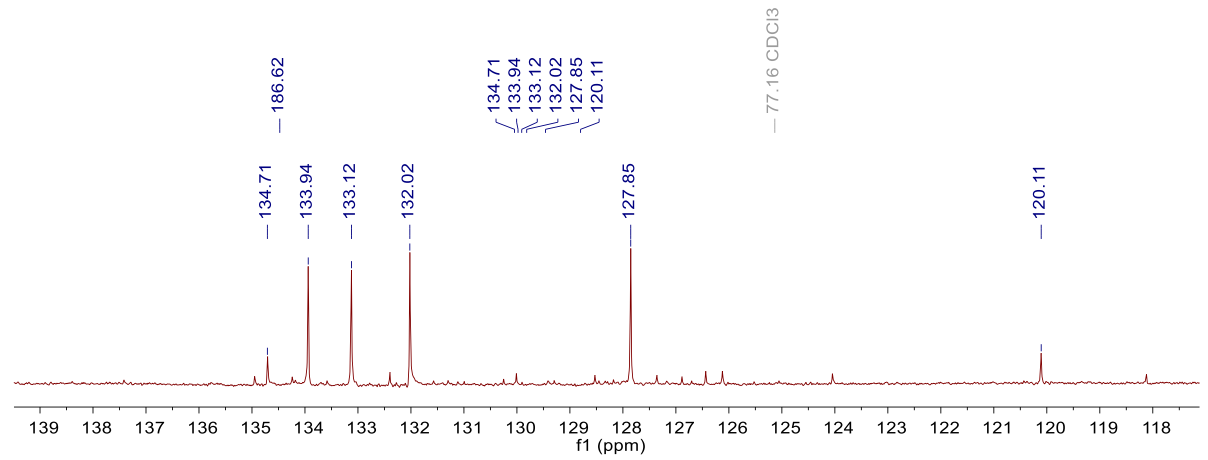
186.62
134.71
133.94
133.12
132.02
127.85
120.11
77.16 CDCl3
f1 (ppm)

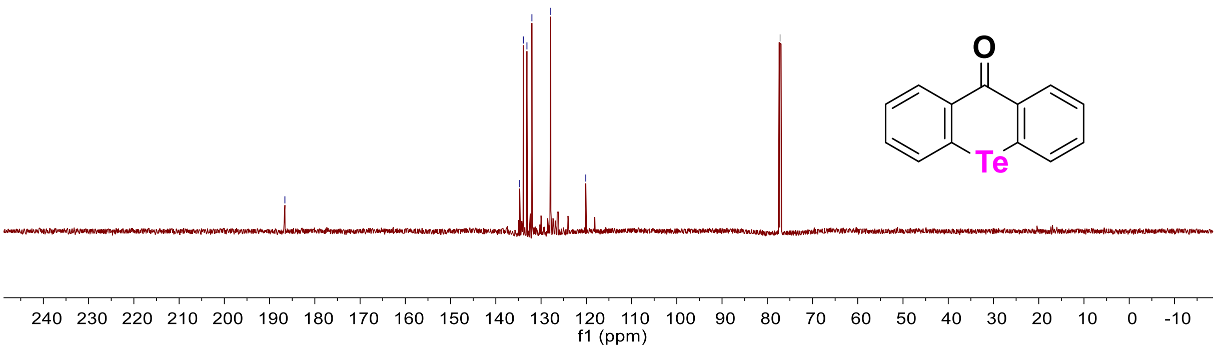
O
Te
f1 (ppm)

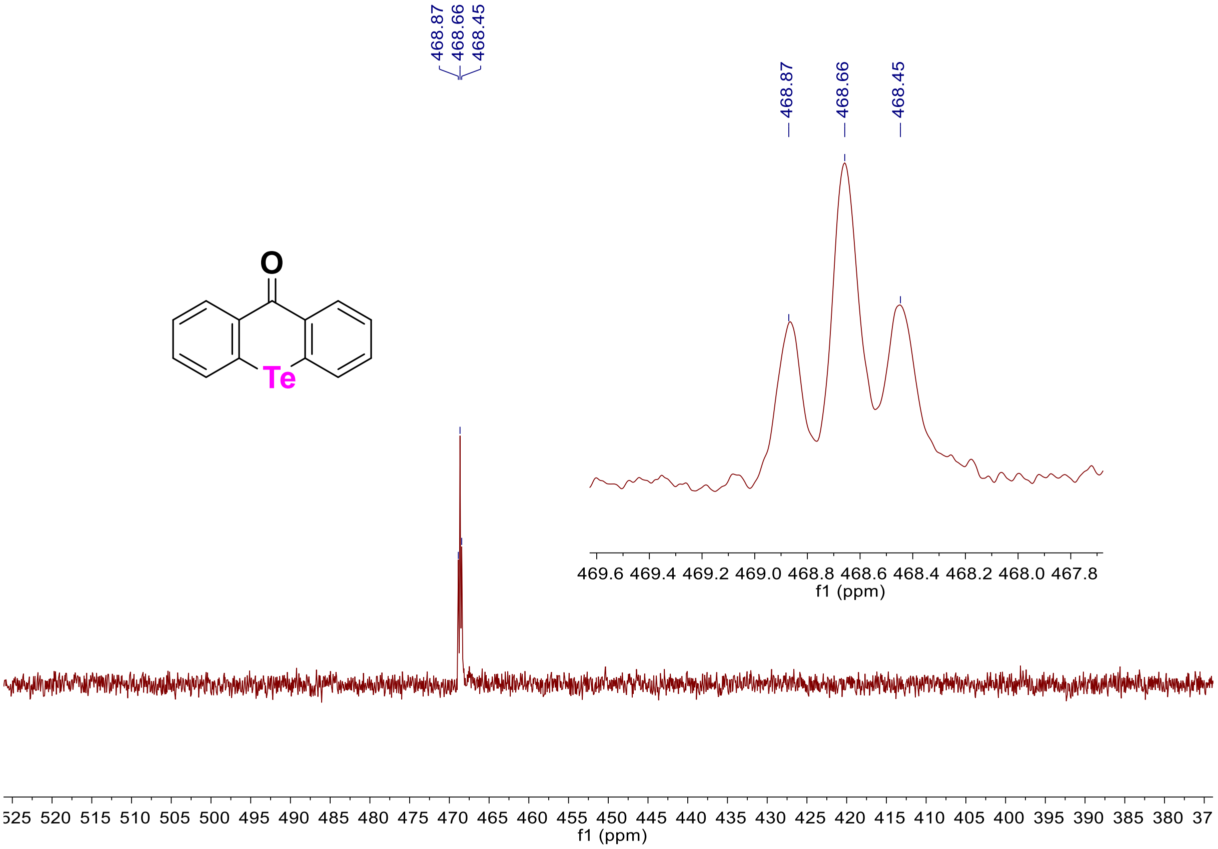
468.87
468.66
468.45
O
Te
f1 (ppm)

9-Phenyl-9*H*-telluroxanthrene Te7

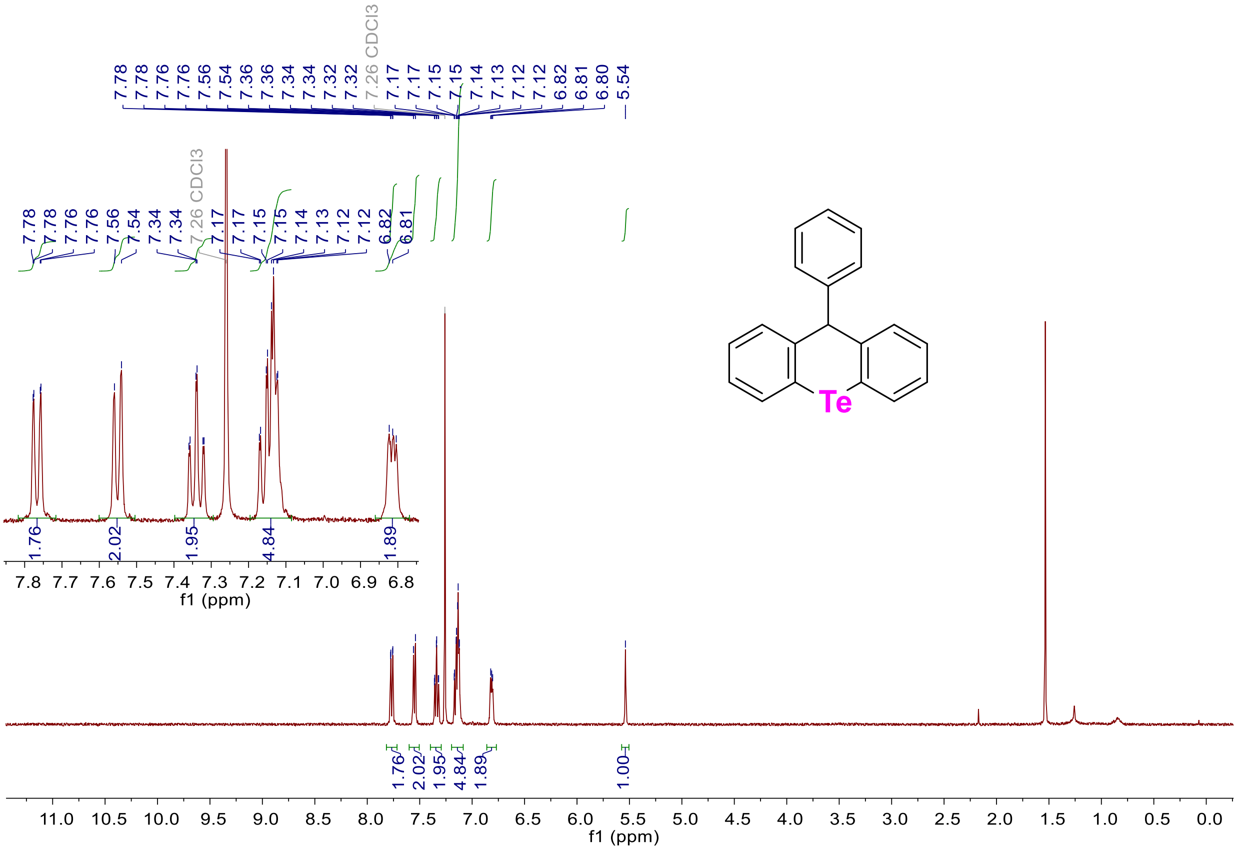

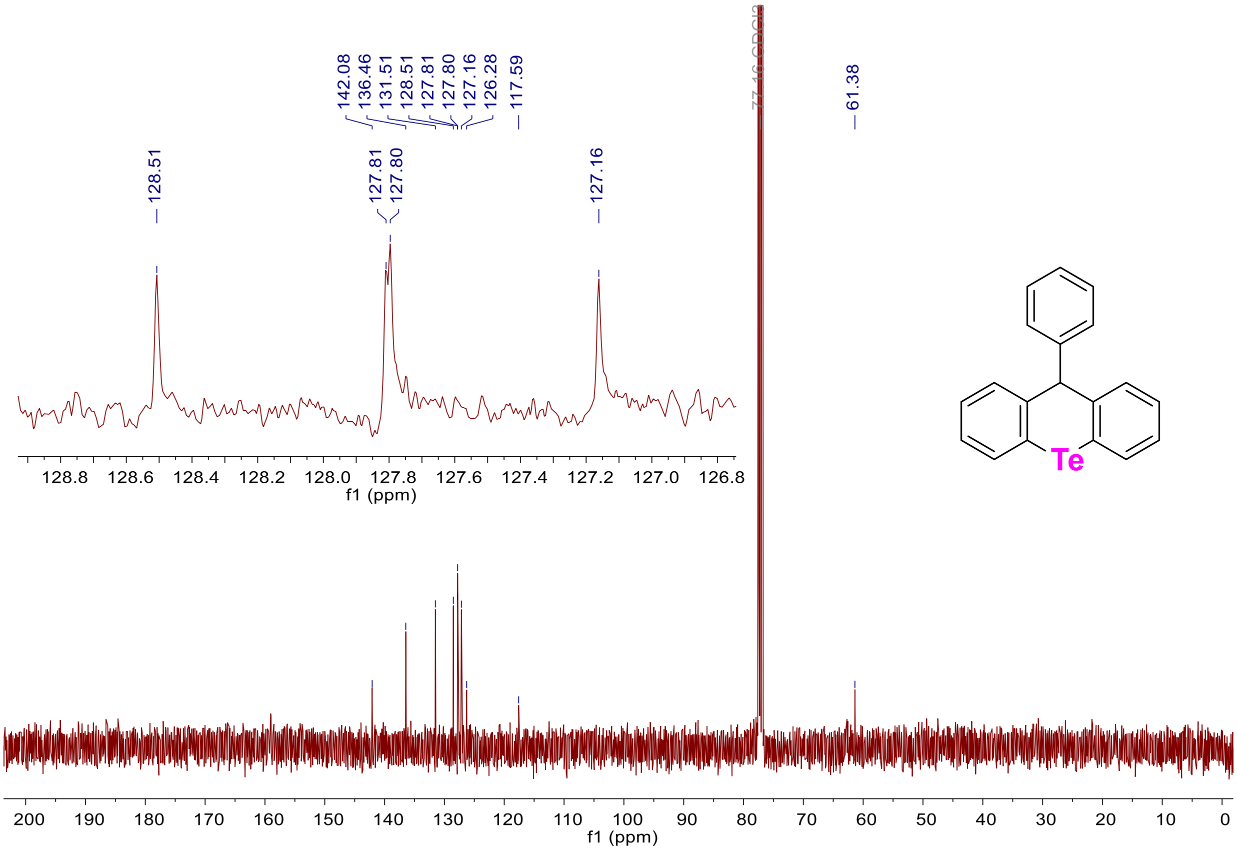

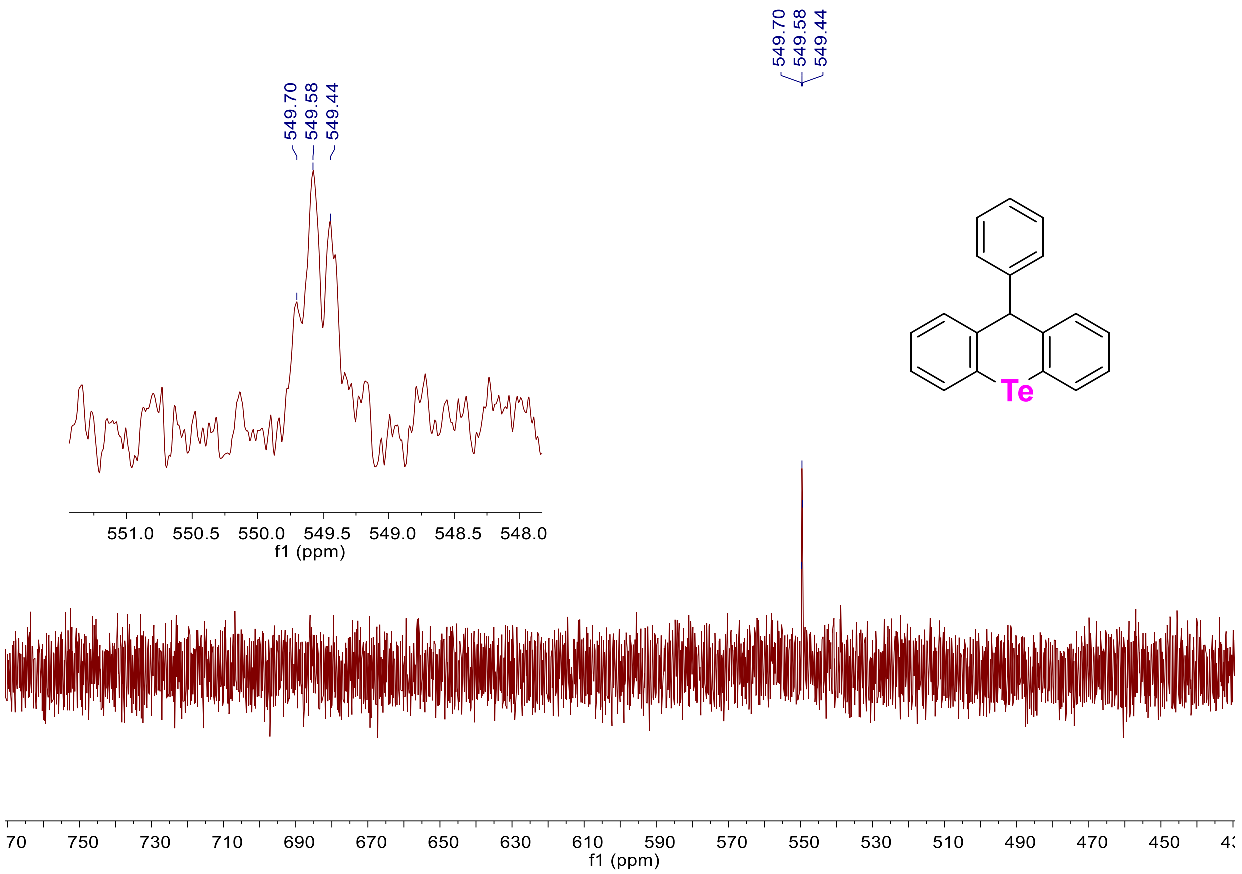

2,8-Dimethoxy-10*H*-phenotellurazine TeZ15

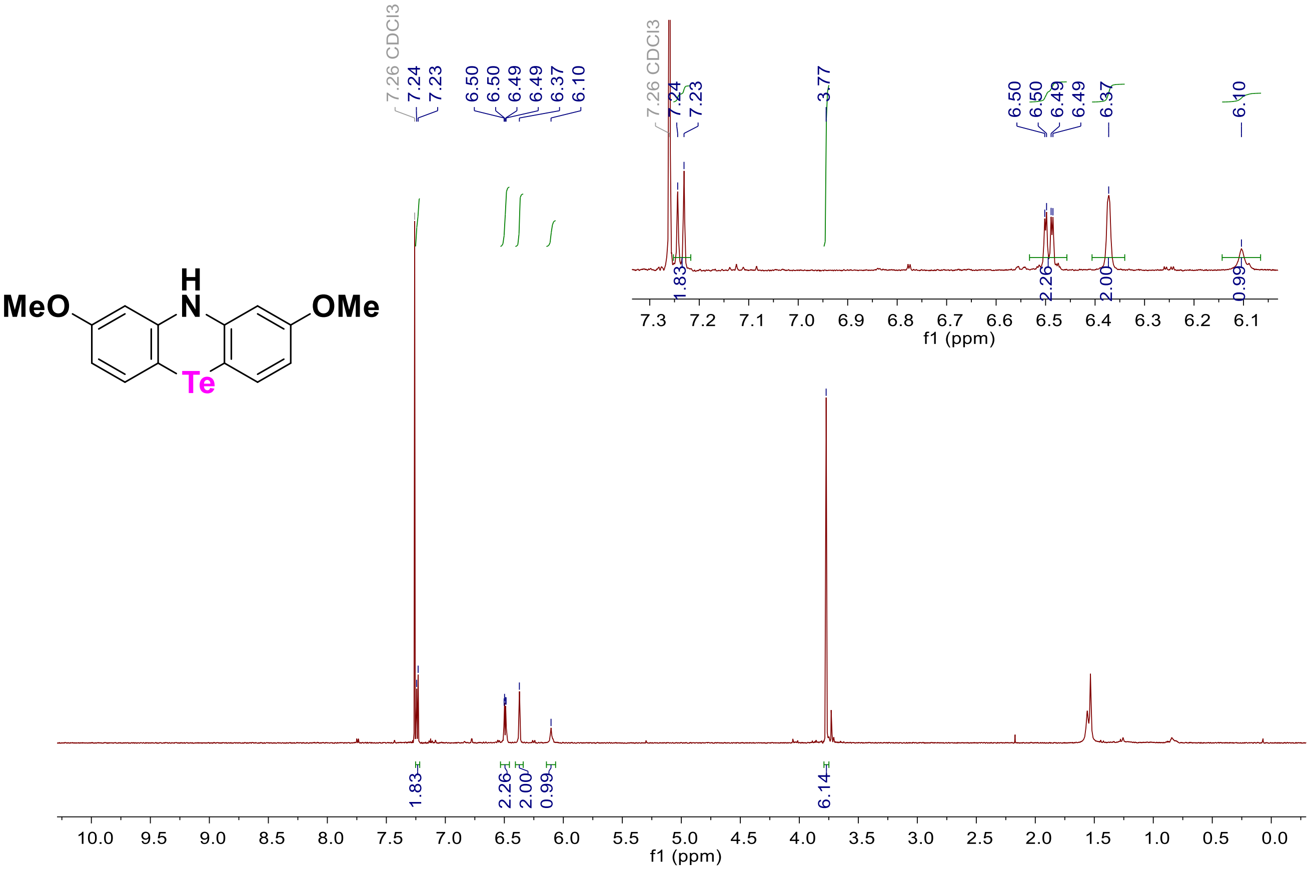

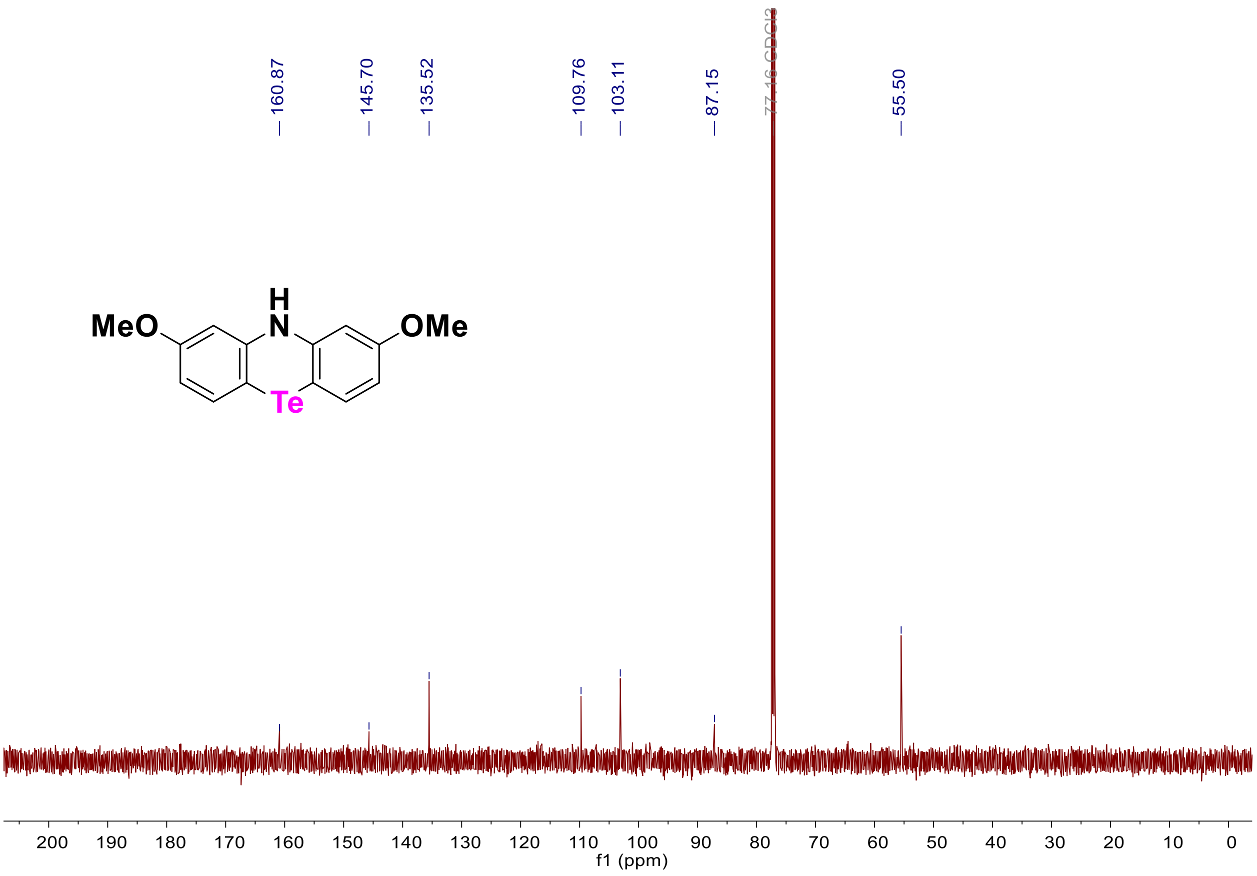
160.87
145.70
135.52
109.76
103.11
87.15
77.16 CDCl3
55.50
MeO
H
N
OMe
Te
200 190 180 170 160 150 140 130 120 110 100 90 80 70 60 50 40 30 20 10 0
f1 (ppm)

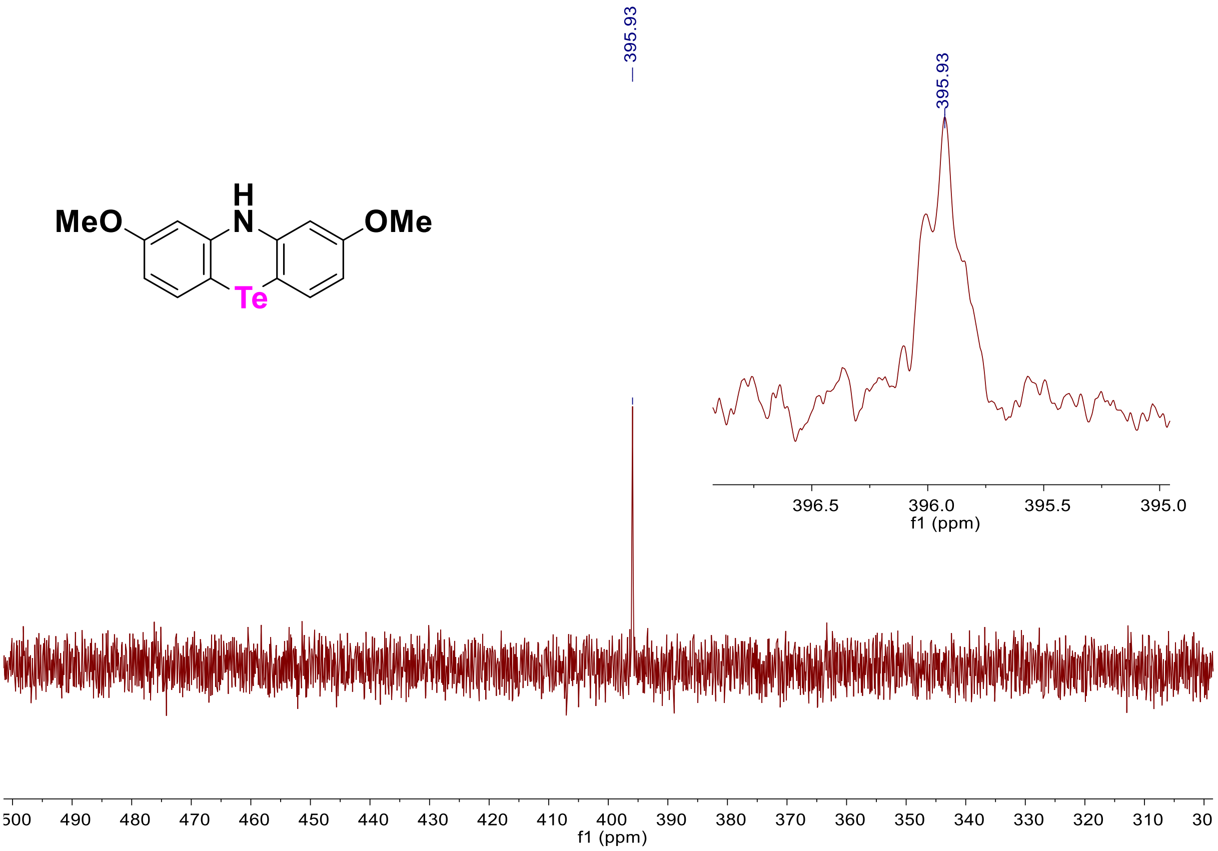
395.93
395.93
MeO
H
N
OMe
Te
396.5 396.0 395.5 395.0
f1 (ppm)
500 490 480 470 460 450 440 430 420 410 400 390 380 370 360 350 340 330 320 310 30
f1 (ppm)

2-*N*,*N*-Dimethylamino-10*H*-phenotellurazine PTeZ16

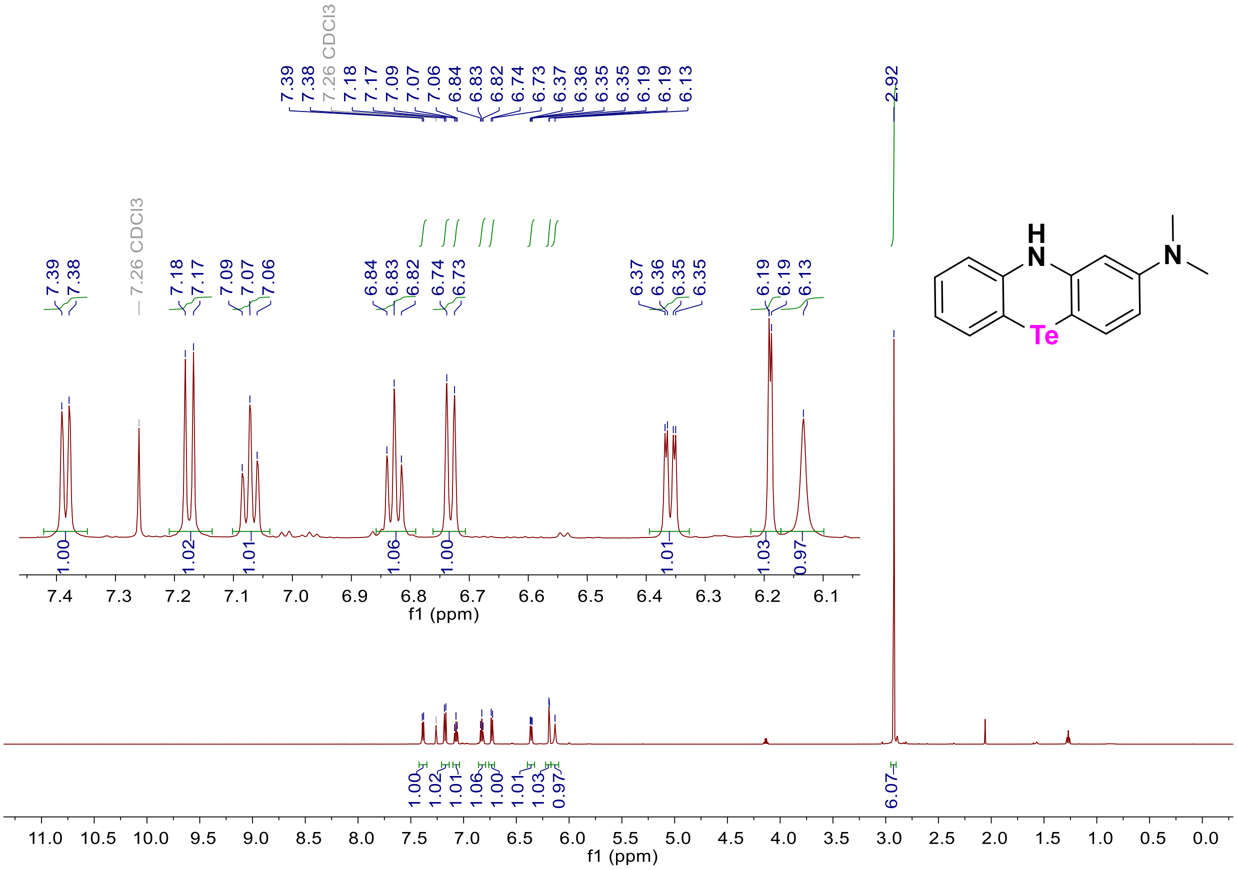

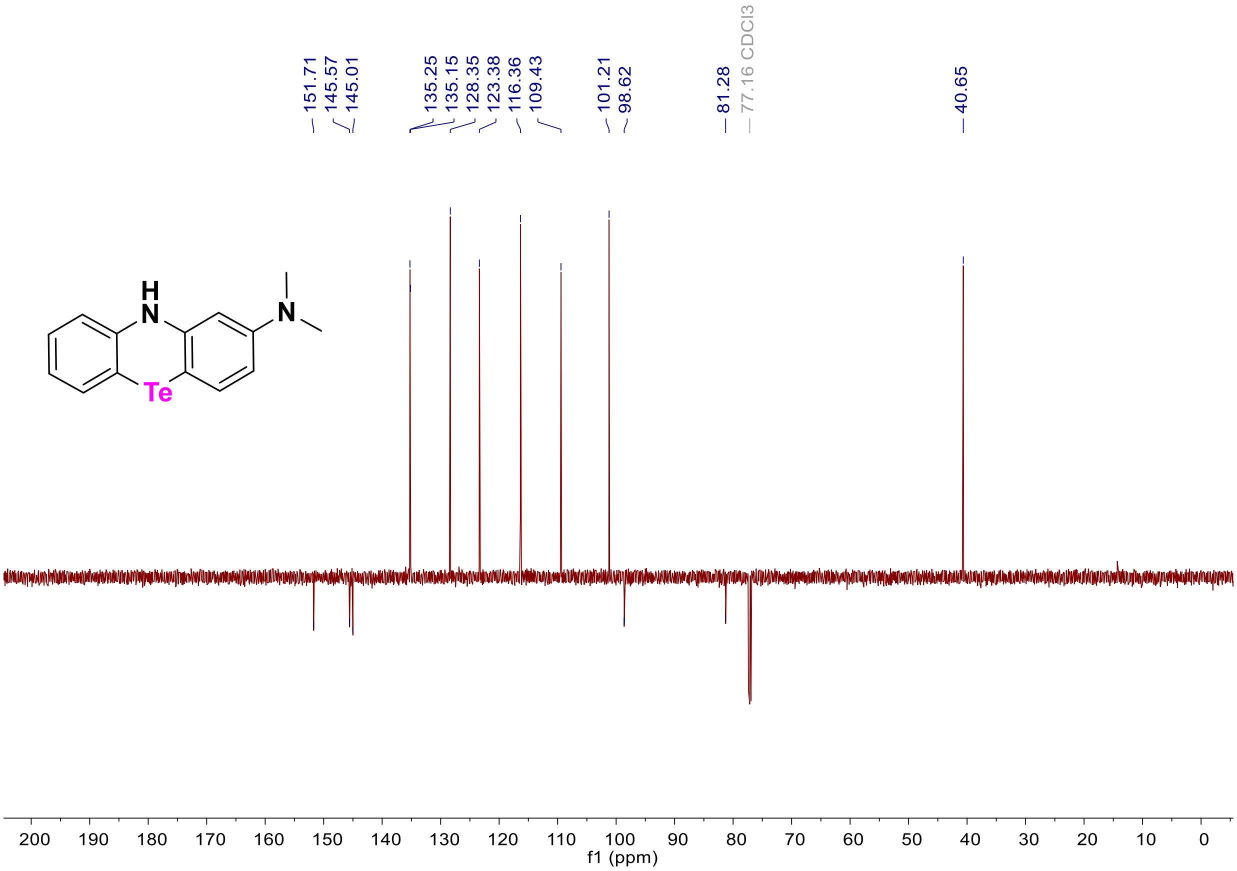

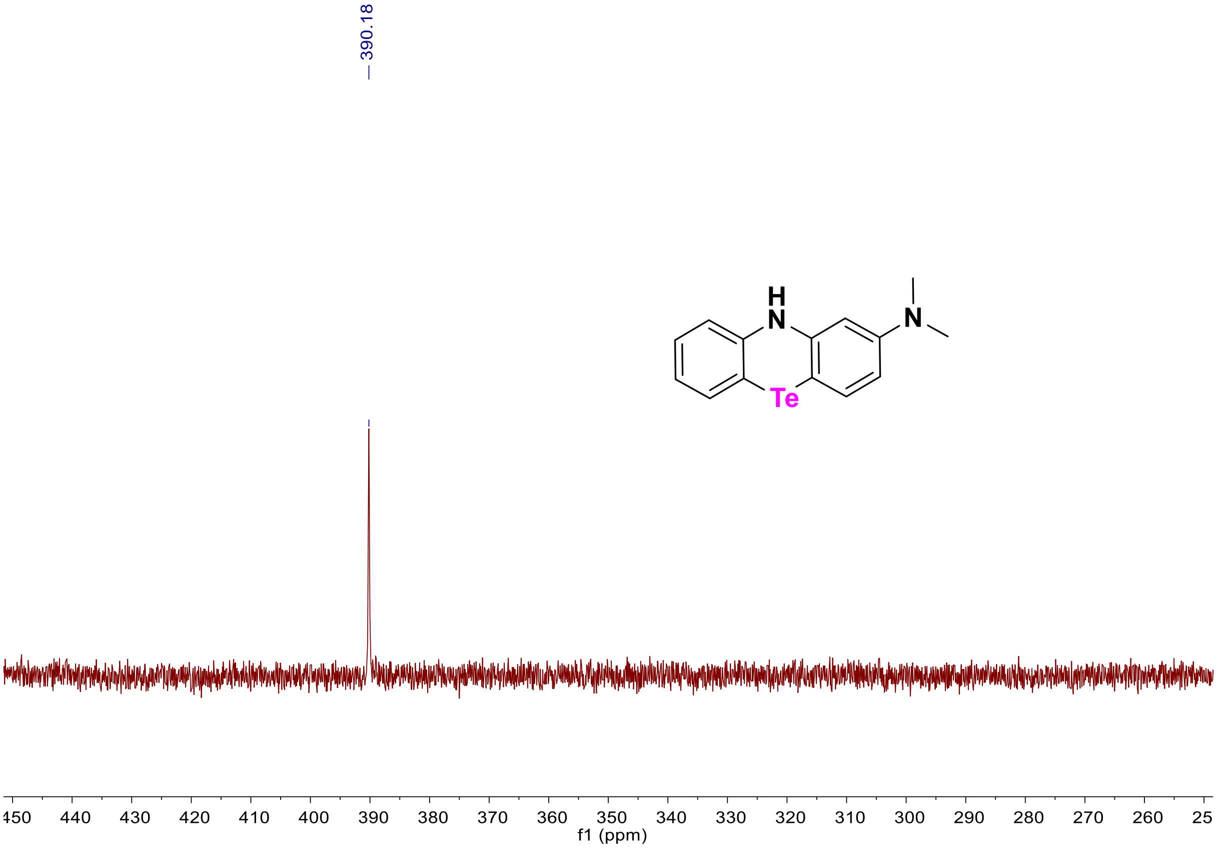

Diphenyltelluride Te8

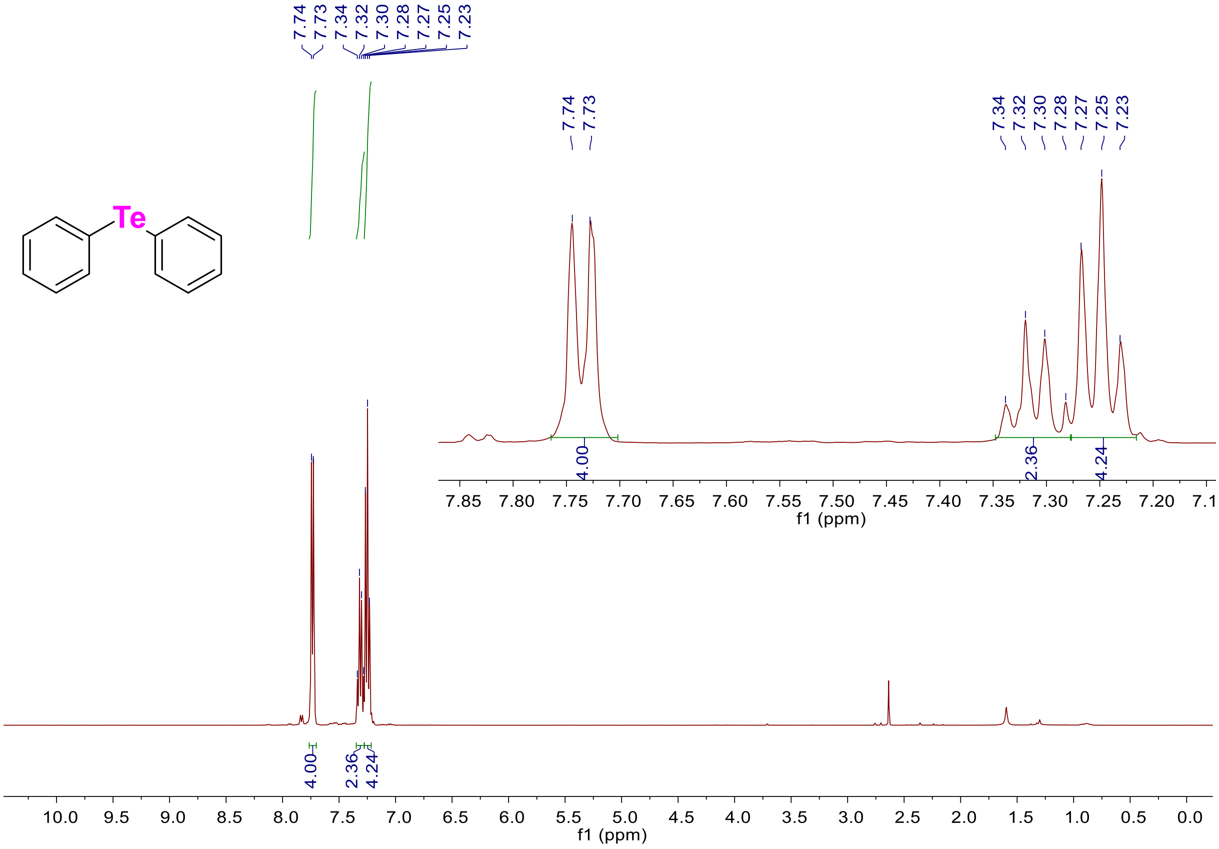

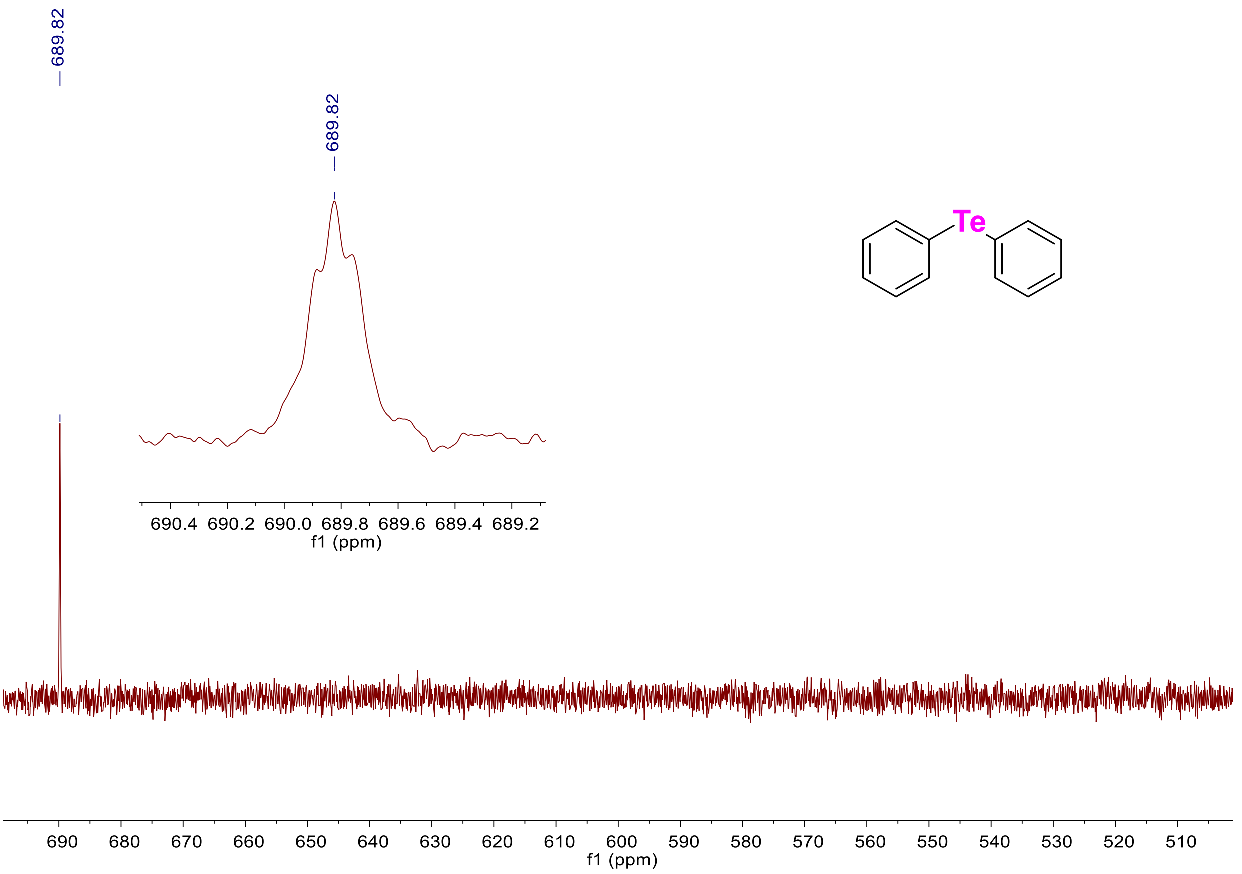

(4-Methoxyphenyl)phenyltellane Te9

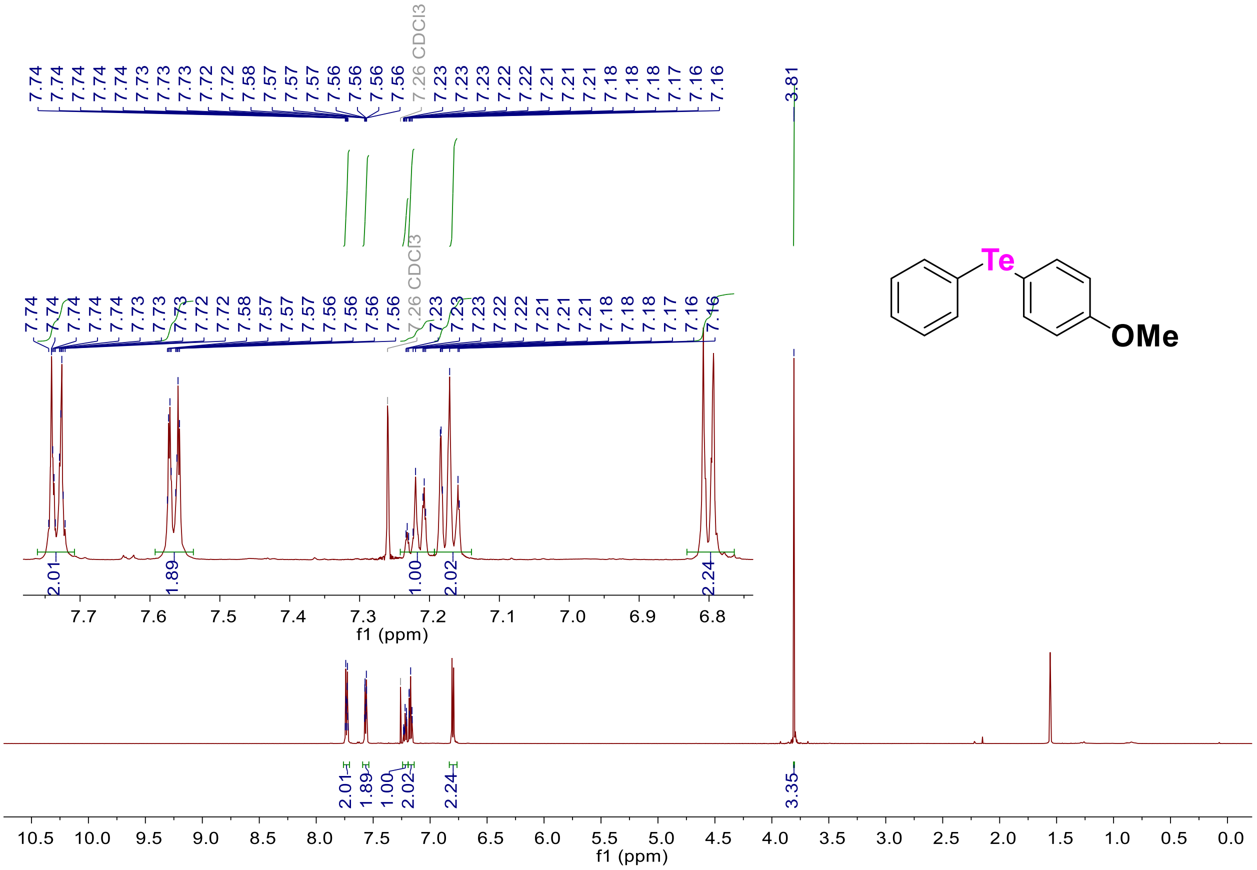

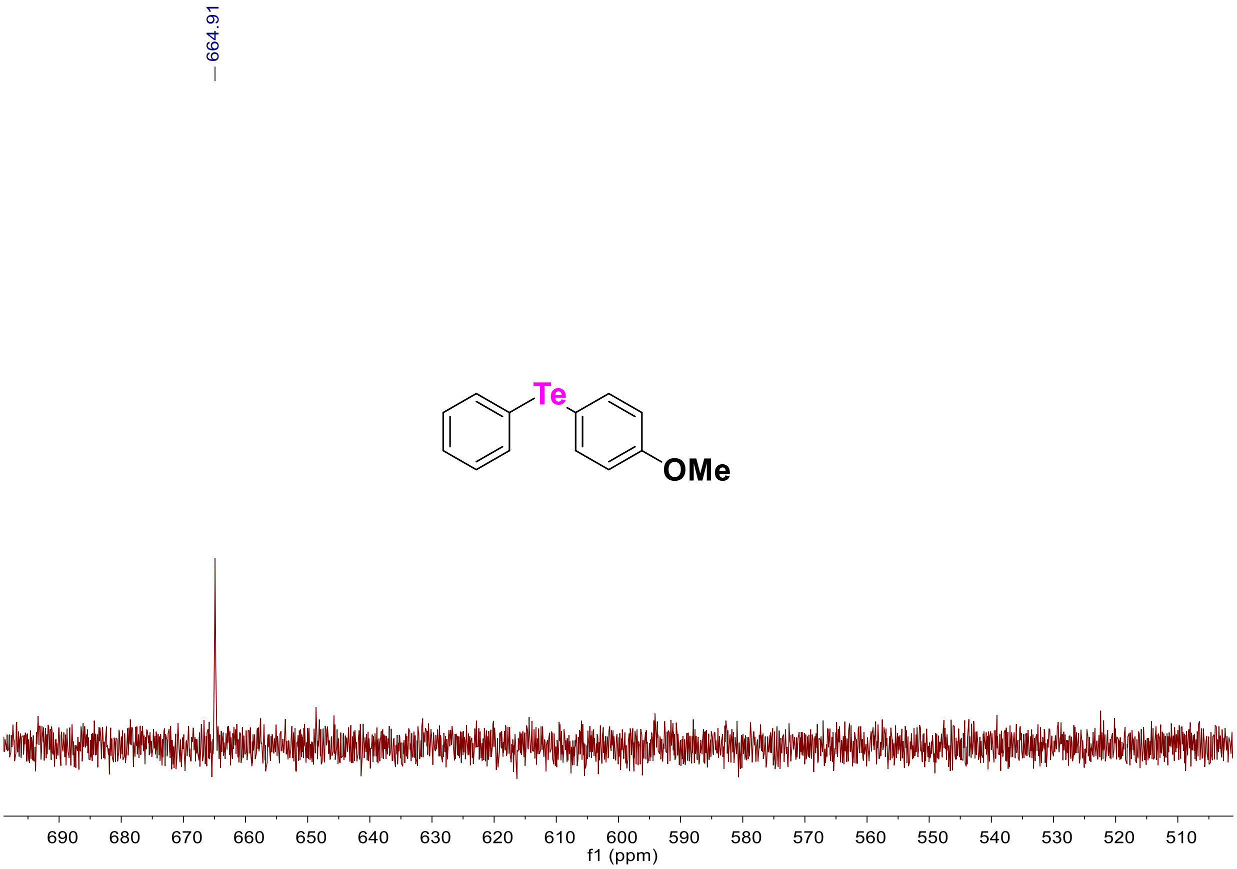

_N,N_-Dimethyl-4-(phenyltellanyl)aniline Te10

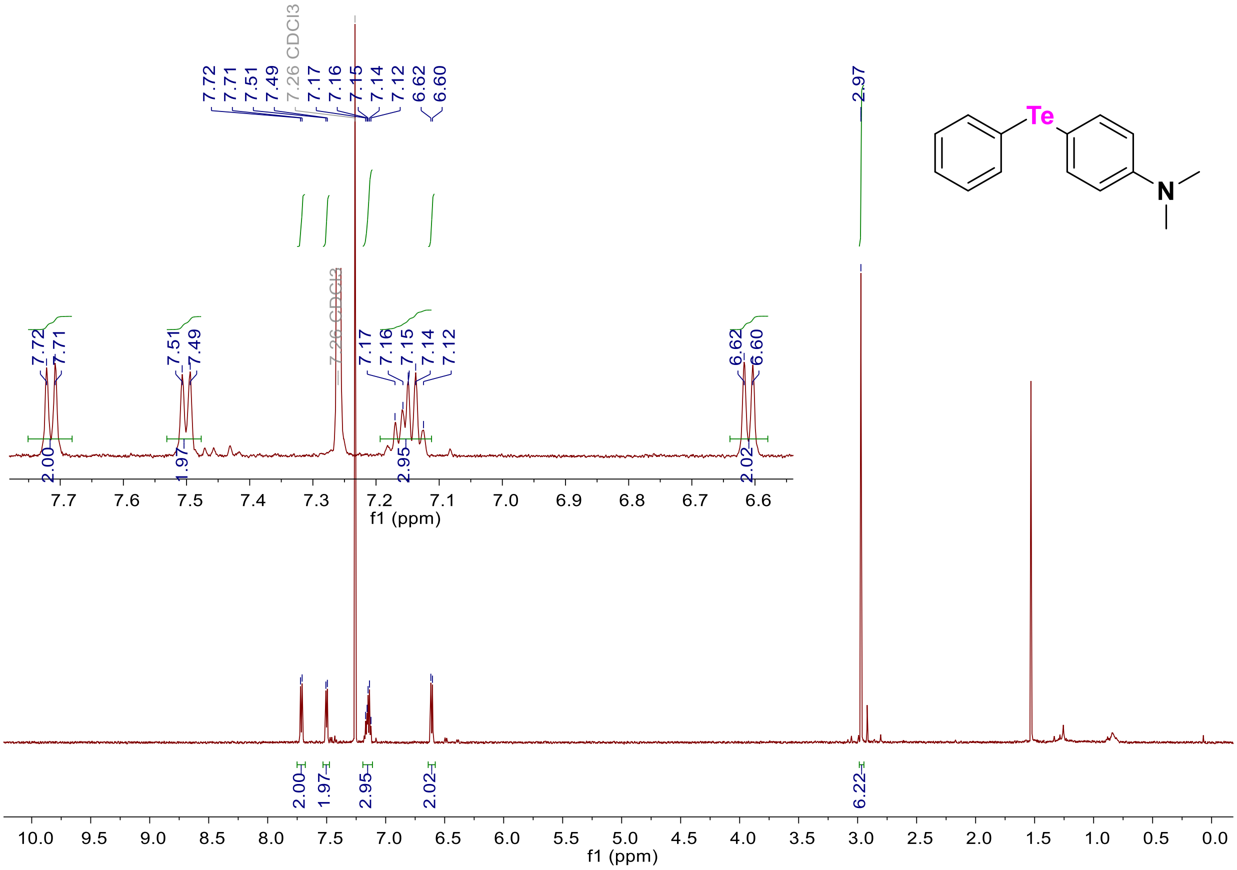

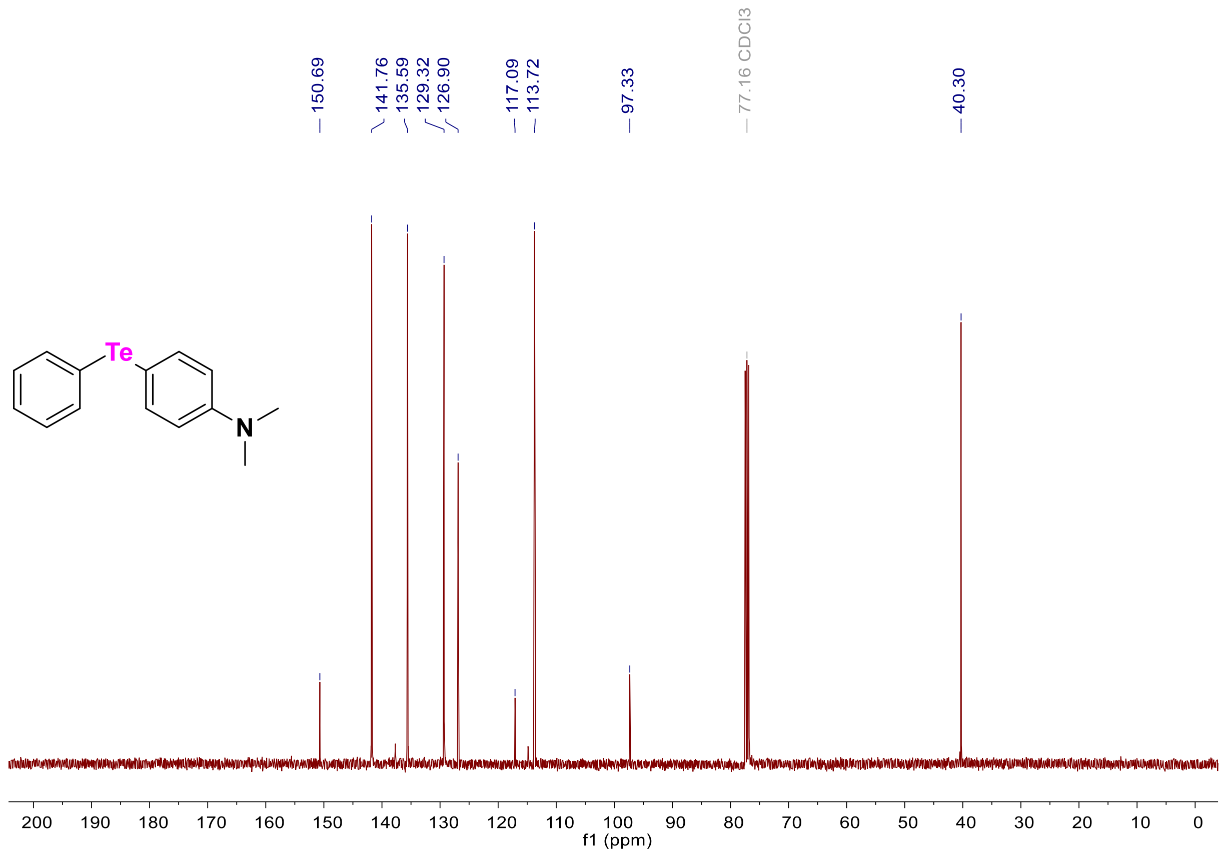
150.69
141.76
135.59
129.32
126.90
117.09
113.72
97.33
77.16 CDCl3
40.30
Te
N
200 190 180 170 160 150 140 130 120 110 100 90 80 70 60 50 40 30 20 10 0
f1 (ppm)

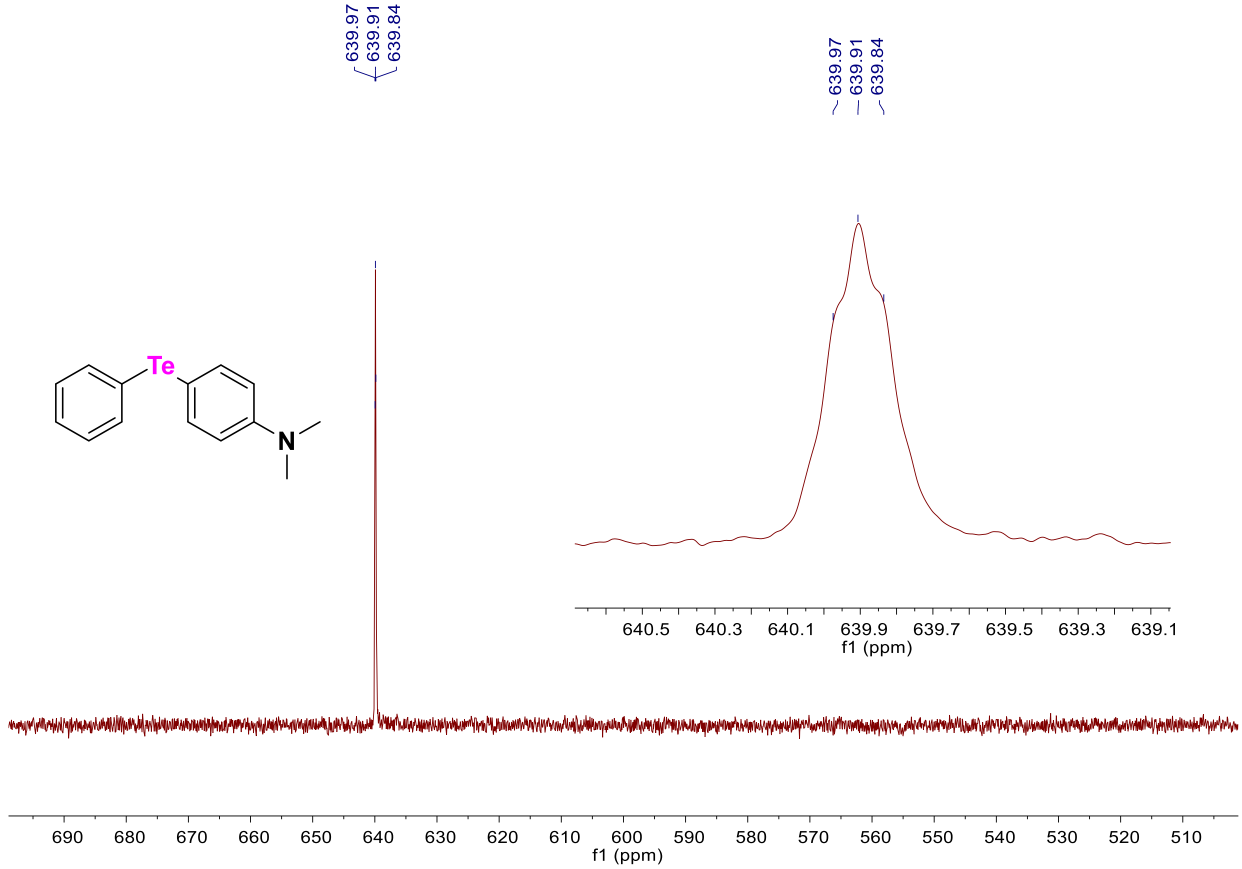
639.97
639.91
639.84
639.97
639.91
639.84
Te
N
640.5 640.3 640.1 639.9 639.7 639.5 639.3 639.1
f1 (ppm)
690 680 670 660 650 640 630 620 610 600 590 580 570 560 550 540 530 520 510
f1 (ppm)

(2,4-Dimethoxyphenyl)phenyltellane Te11

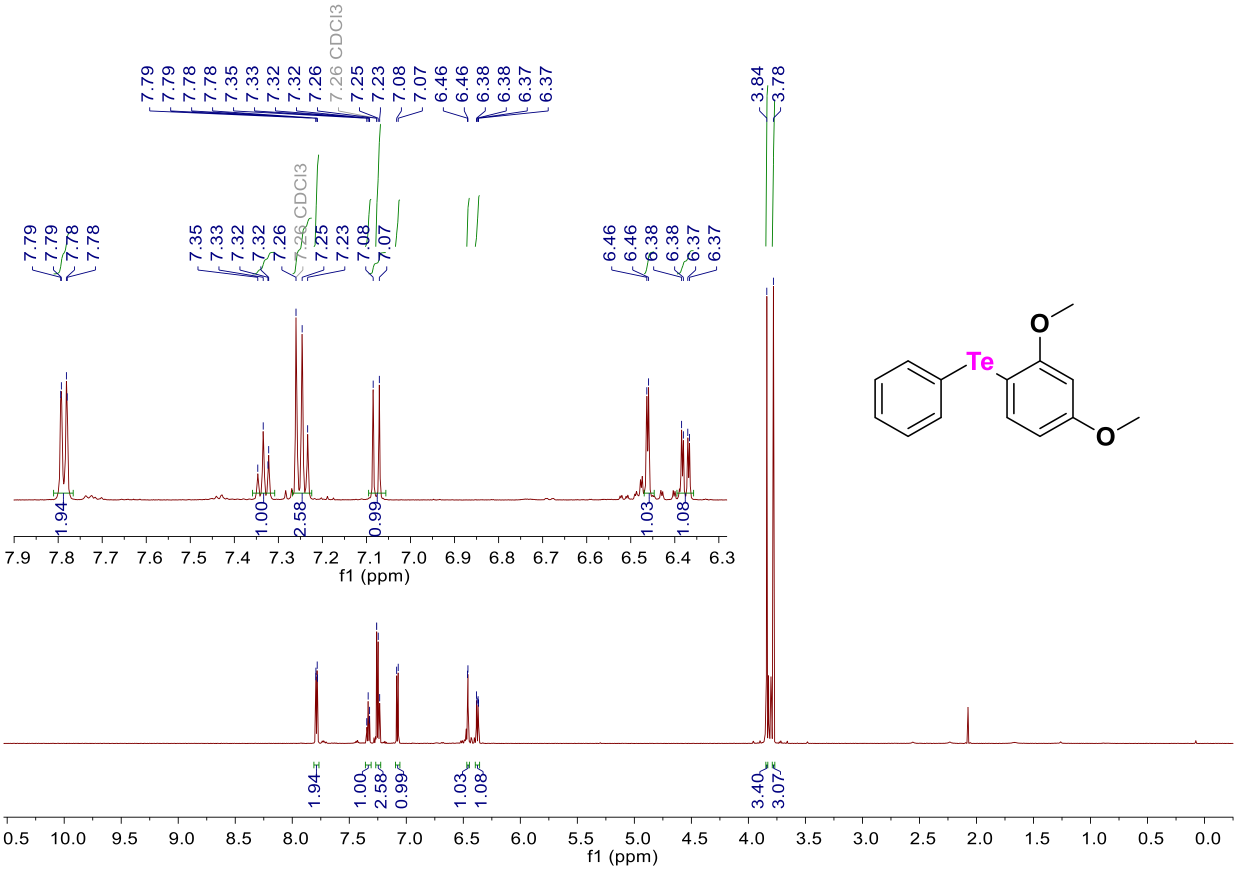

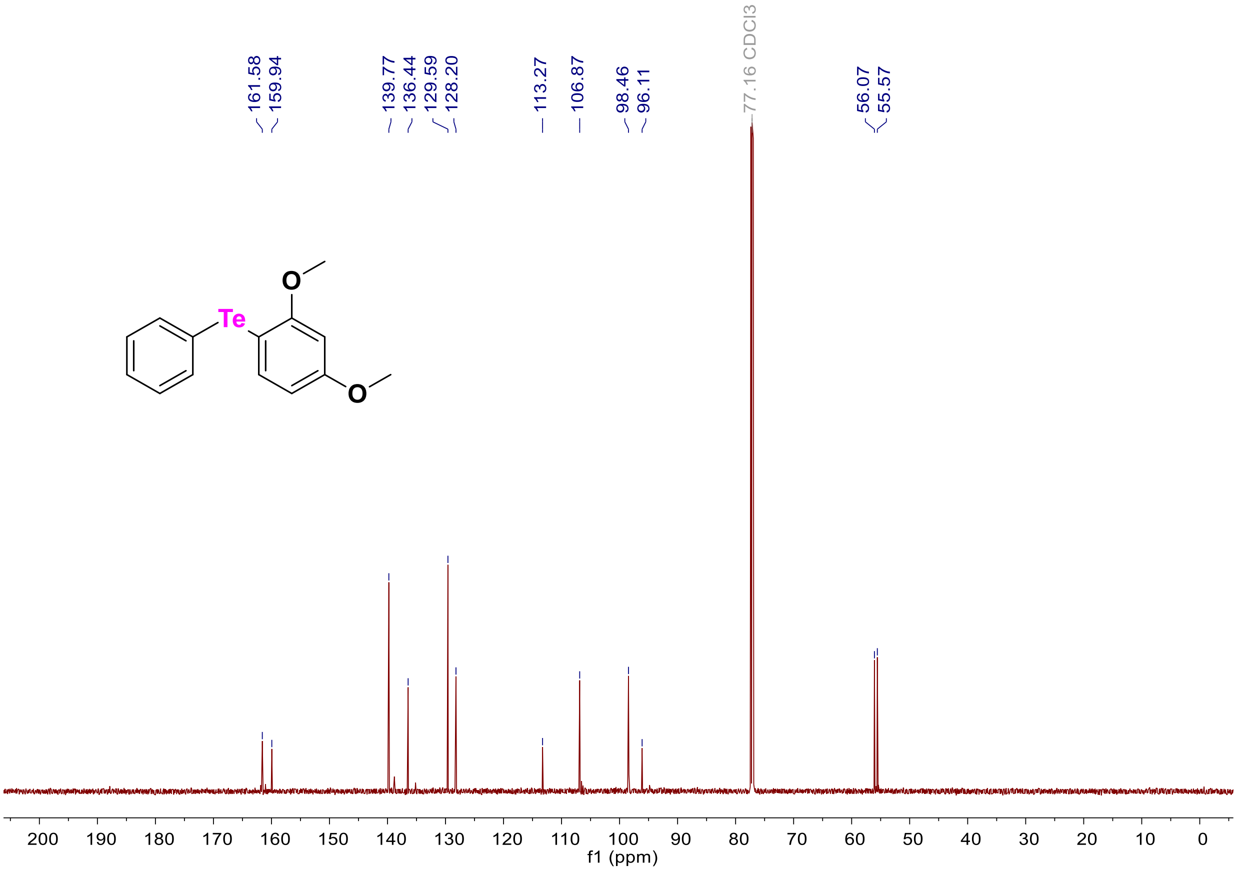

(3,5-Dimethoxyphenyl)phenyltellane Te12

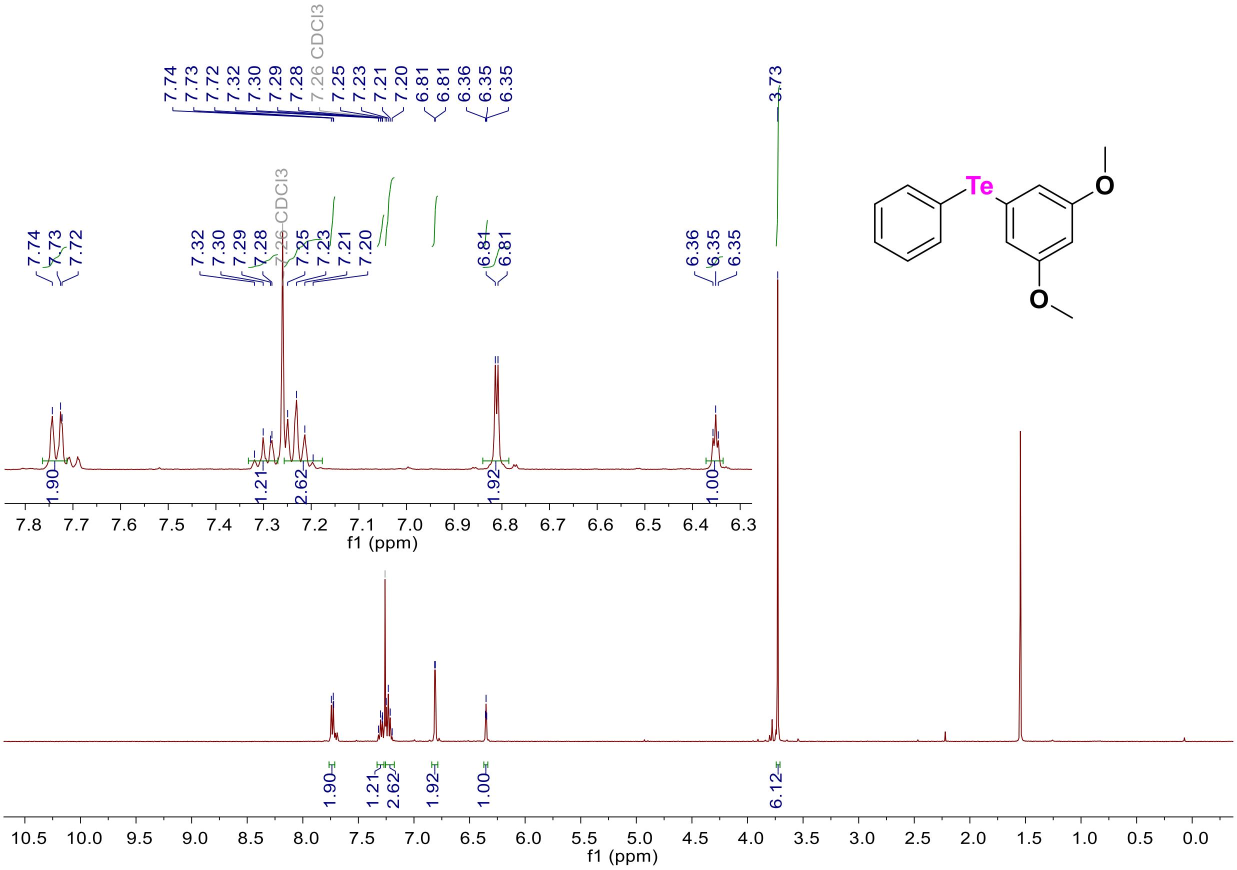

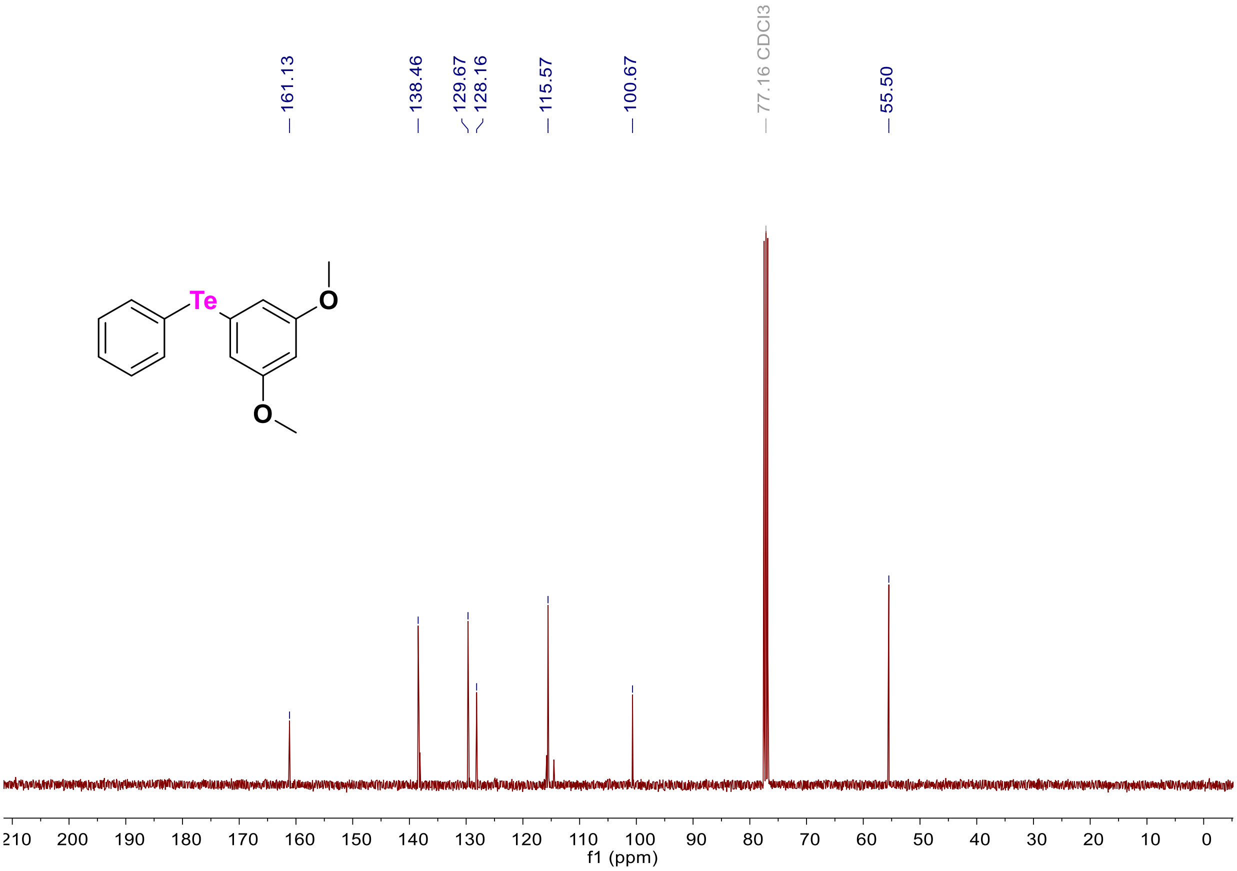

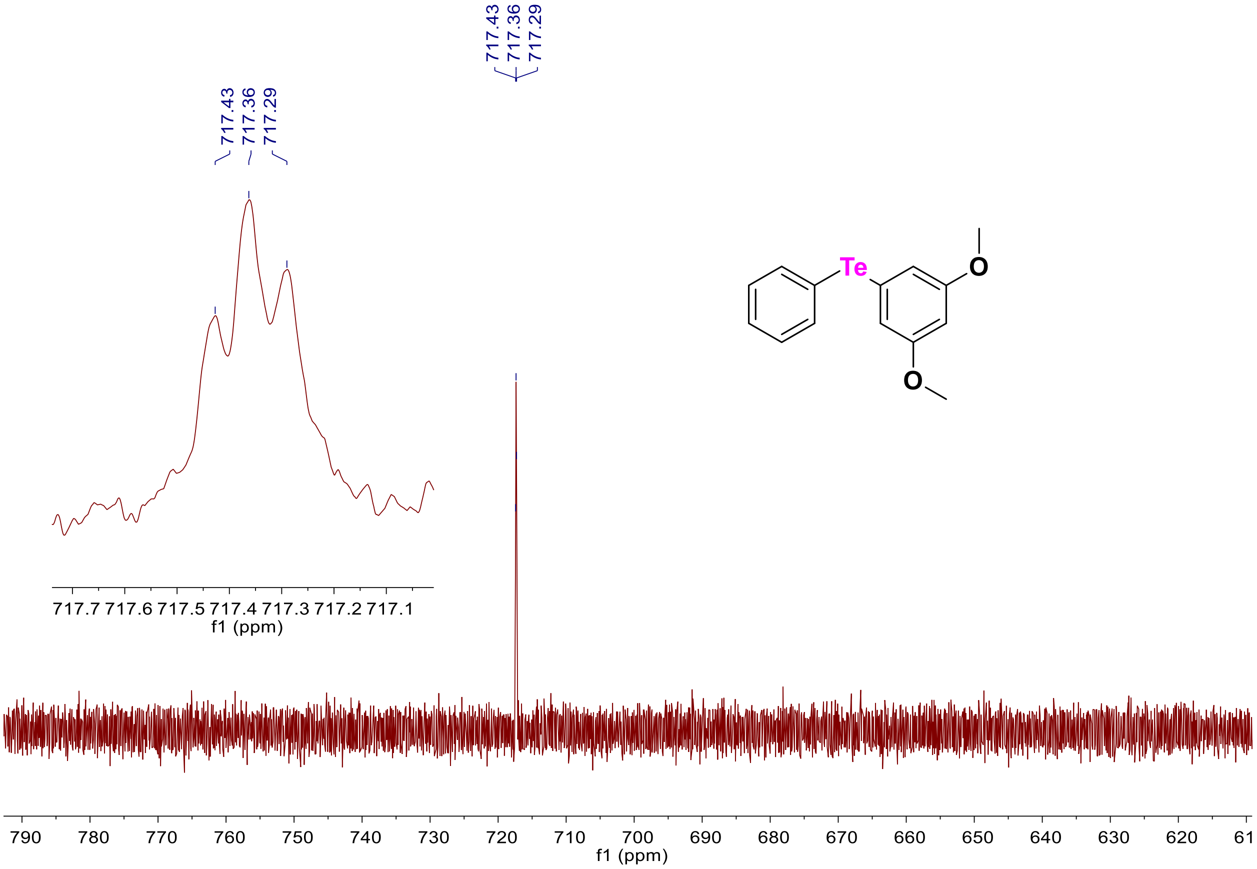

(3,4,5-Trimethoxyphenyl)phenyltellane Te13

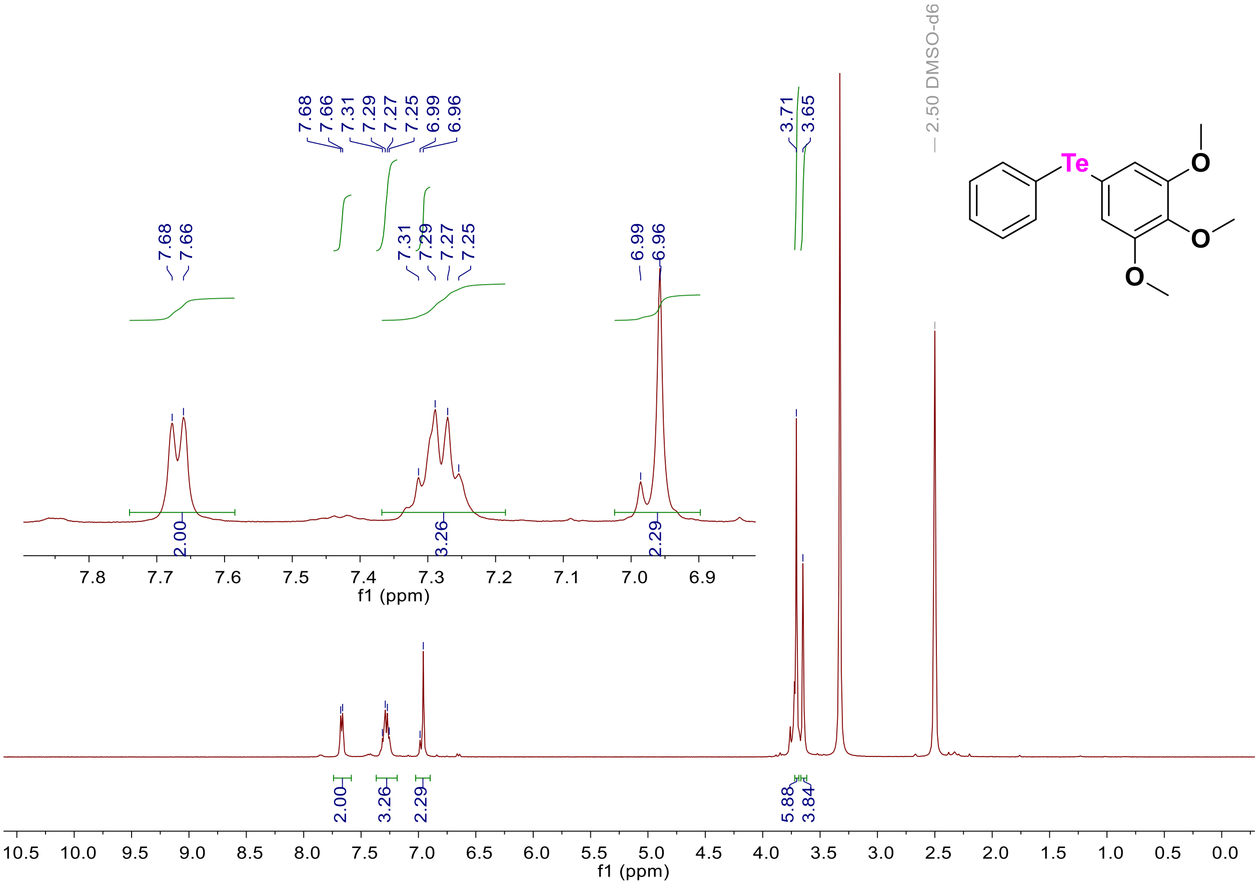

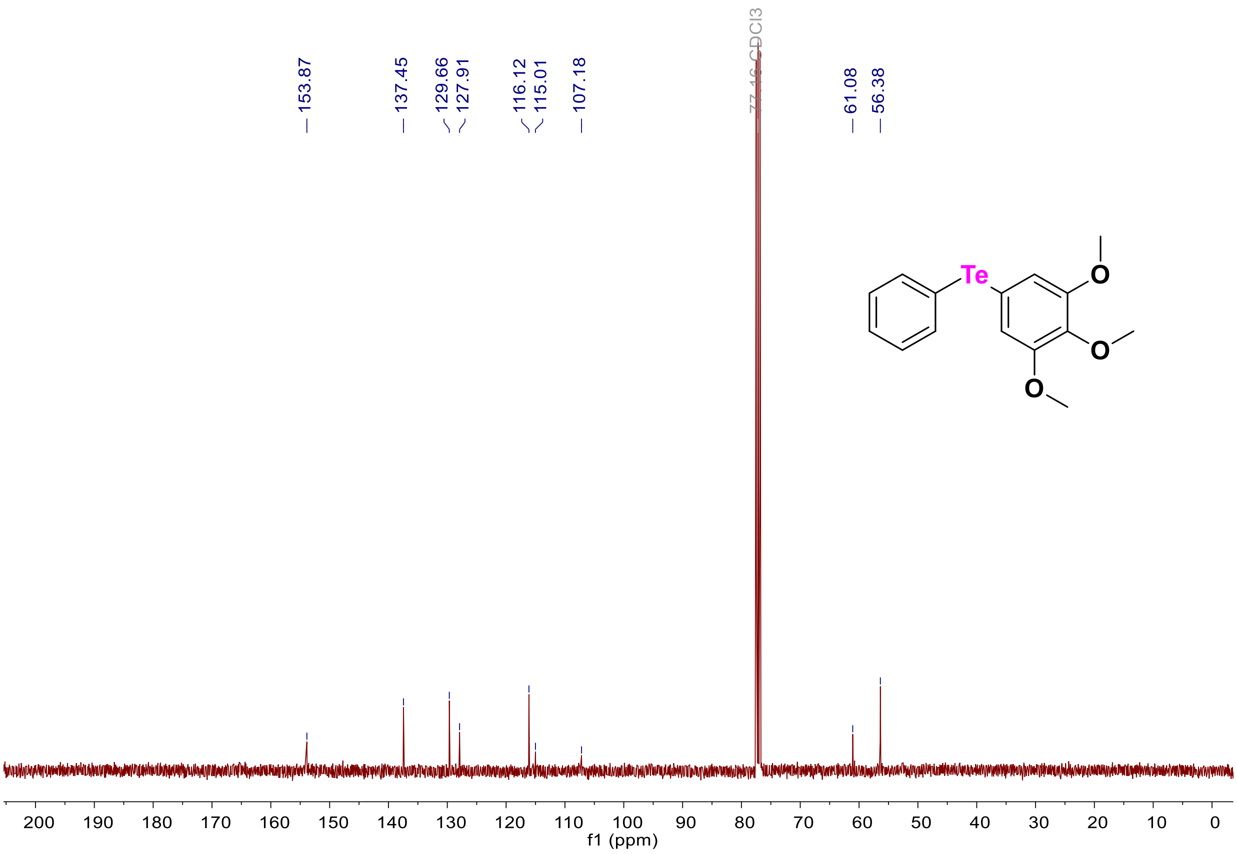
153.87
137.45
129.66
127.91
116.12
115.01
107.18
77.16 CDCl3
61.08
56.38
Te
O
O
O
200 190 180 170 160 150 140 130 120 110 100 90 80 70 60 50 40 30 20 10 0
f1 (ppm)

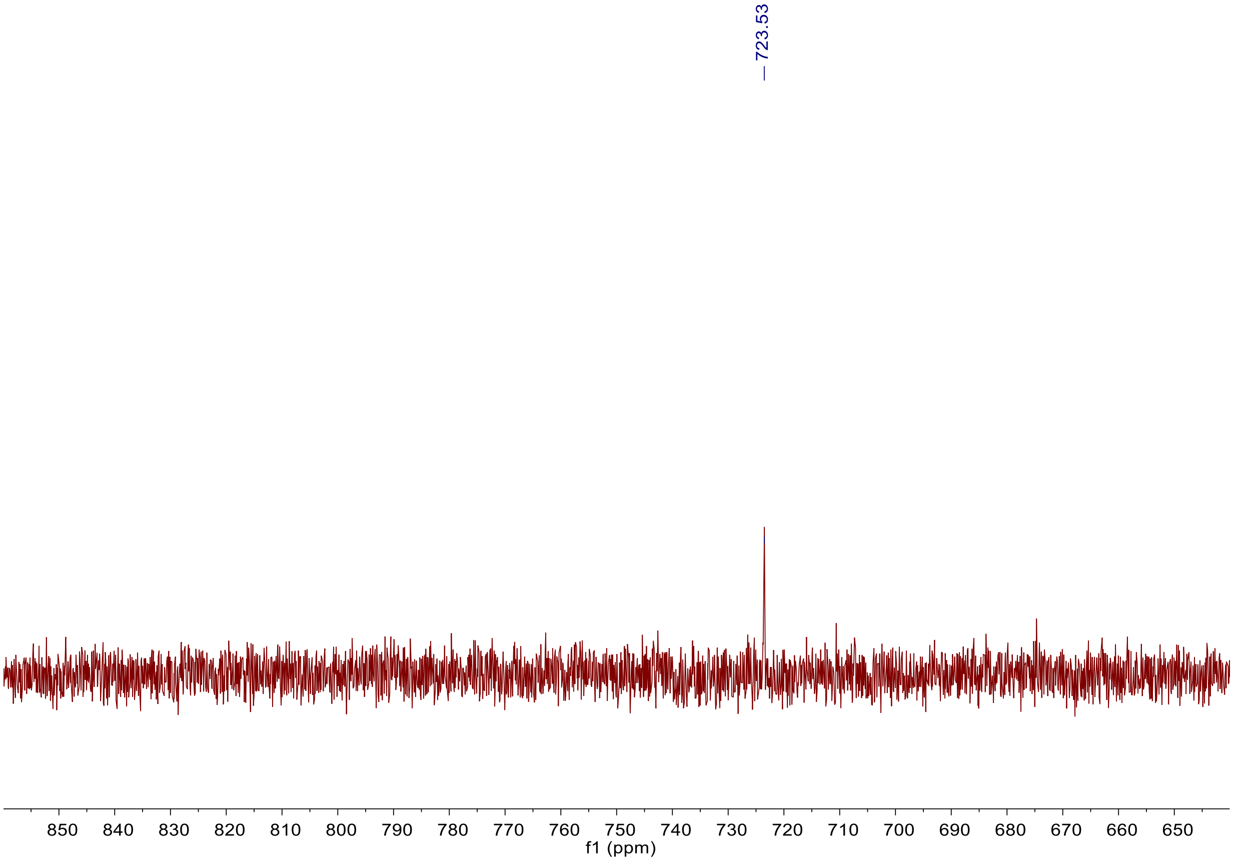
723.53
850 840 830 820 810 800 790 780 770 760 750 740 730 720 710 700 690 680 670 660 650
f1 (ppm)

N-(2-Hydroxy-5-phenylphenyl)-10H-phenothiazine 3aa

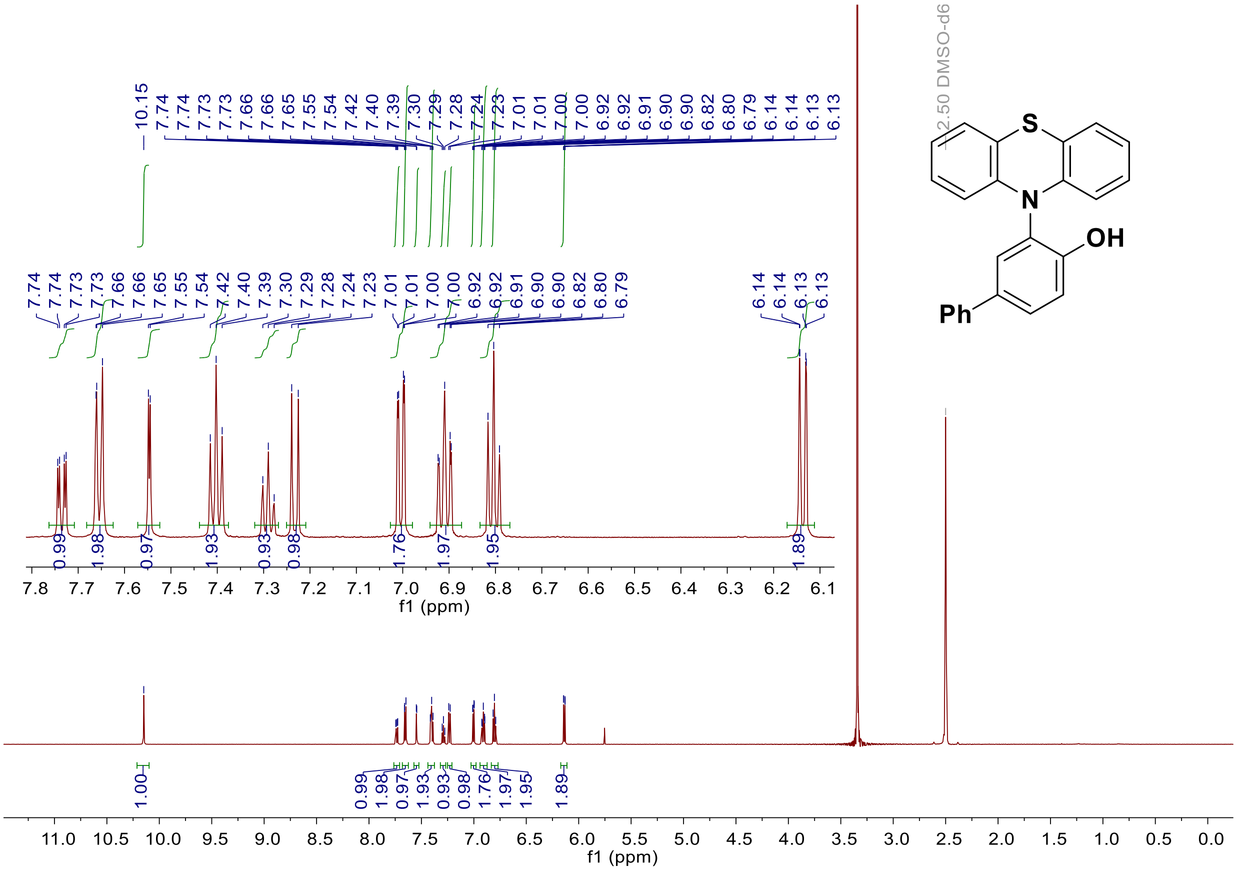

2-Phenyl-2-(2-phenyl-1H-indol-3-yl)indolin-3-one 5a

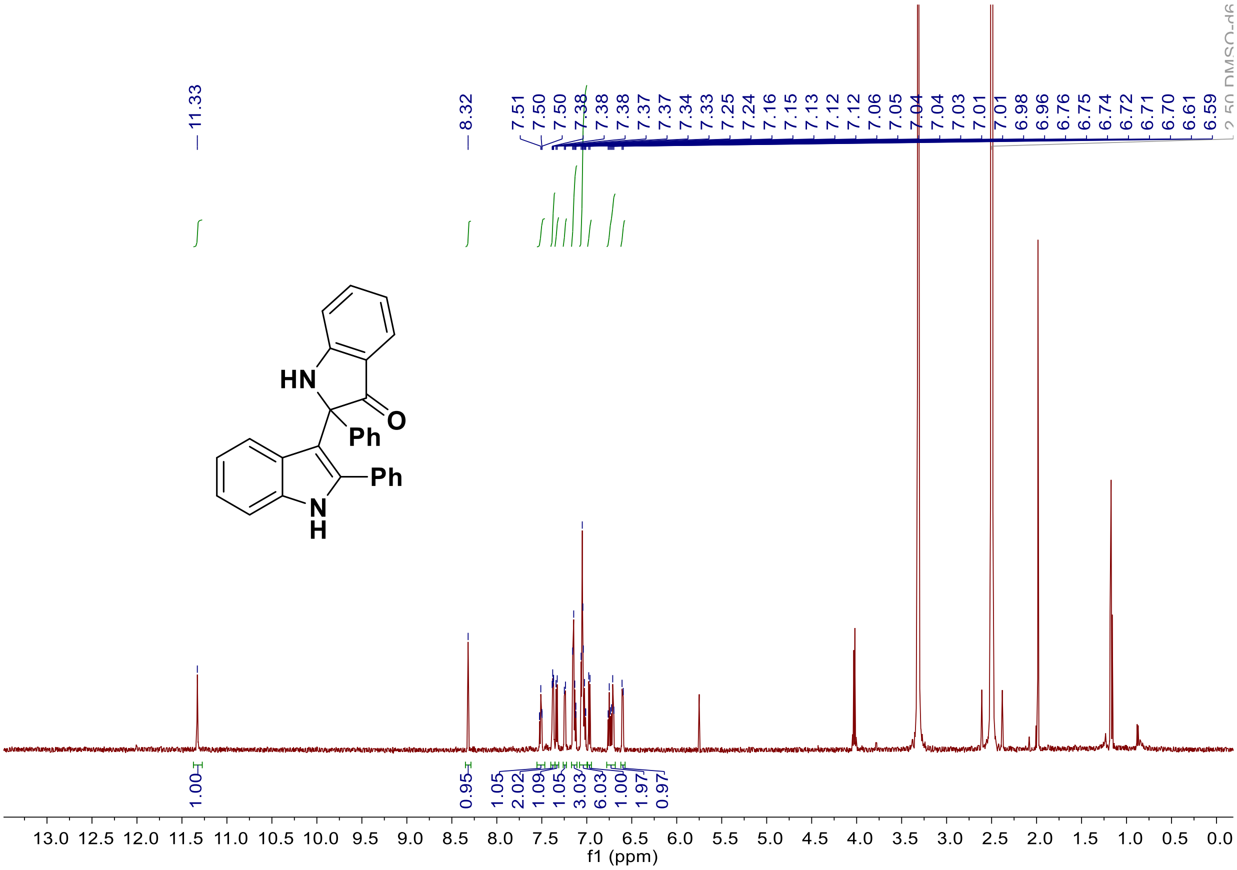

Supplement: File 1 — Experimental section and characterization of synthesized compounds. [file Beilstein_J_Org_Chem-20-1292-s001.pdf]
